# E3 ligase activity of Carboxyl terminus of Hsc70 interacting protein (CHIP) in Wharton's jelly derived mesenchymal stem cells improves their persistence under hyperglycemic stress and promotes the prophylactic effects against diabetic cardiac damages

**DOI:** 10.1002/btm2.10234

**Published:** 2021-06-11

**Authors:** Ayaz Ali, Wei‐Wen Kuo, Chia‐Hua Kuo, Jeng‐Fan Lo, Michael Y. C. Chen, Jayasimha R. Daddam, Tsung‐Jung Ho, Vijaya Padma Viswanadha, Marthandam Asokan Shibu, Chih‐Yang Huang

**Affiliations:** ^1^ Department of Biological Science and Technology China Medical University Taichung Taiwan; ^2^ Ph.D. Program for Biotechnology Industry, China Medical University Taichung Taiwan; ^3^ Laboratory of Exercise Biochemistry University of Taipei Taipei Taiwan; ^4^ Institute of Oral Biology, National Yang‐Ming University Taipei Taiwan; ^5^ Department of Cardiology Buddhist Tzu Chi General Hospital Hualien Taiwan; ^6^ Cardiovascular and Mitochondrial Related Disease Research Center, Hualien Tzu Chi Hospital, Buddhist Tzu Chi Medical Foundation Hualien Taiwan; ^7^ Department of Chinese Medicine Hualien Tzu Chi Hospital, Buddhist Tzu Chi Medical Foundation, Tzu Chi University Hualien Taiwan; ^8^ Integration Center of Traditional Chinese and Modern Medicine, Hualien Tzu Chi Hospital, Buddhist Tzu Chi Medical Foundation Hualien Taiwan; ^9^ Department of Biotechnology Bharathiar University Coimbatore India; ^10^ Graduate Institute of Biomedical Sciences, China Medical University Taichung Taiwan; ^11^ Department of Medical Research China Medical University Hospital, China Medical University Taichung Taiwan; ^12^ Department of Biotechnology Asia University Taichung Taiwan; ^13^ Center of General Education, Buddhist Tzu Chi Medical Foundation Tzu Chi University of Science and Technology Hualien Taiwan

**Keywords:** apoptosis, carboxyl terminus of Hsc70 interacting protein, diabetes, phosphatase and tensin homolog, Wharton's jelly derived mesenchymal stem cells

## Abstract

Recent studies indicate that umbilical cord stem cells are cytoprotective against several disorders. One critical limitation in using stem cells is reduction in their viability under stressful conditions, such as diabetes. However, the molecular intricacies responsible for diabetic conditions are not fully elucidated. In this study, we found that high glucose (HG) conditions induced loss of chaperone homeostasis, stabilized PTEN, triggered the downstream signaling cascade, and induced apoptosis and oxidative stress in Wharton's jelly derived mesenchymal stem cells (WJMSCs). Increased Carboxyl terminus of Hsc70 interacting protein (CHIP) expression promoted phosphatase and tensin homolog (PTEN) degradation via the ubiquitin‐proteasome system and shortened its half‐life during HG stress. Docking studies confirmed the interaction of CHIP with PTEN and FOXO3a with the *Bim* promoter region. Further, it was found that the chaperone system is involved in CHIP‐mediated PTEN proteasomal degradation. CHIP depletion stabilizes PTEN whereas PTEN inhibition showed an inverse effect. CHIP overactivation suppressed the binding of FOXO3a with *bim*. Coculturing CHIP overexpressed WJMSCs suppressed HG‐induced apoptosis and oxidative stress in embryo derived cardiac cell lines. CHIP overexpressing and PTEN silenced WJMSCs ameliorated diabetic effects in streptozotocin (STZ) induced diabetic rats and further improved their body weight and heart weight, and rescued from hyperglycemia‐induced cardiac injury. Considering these, the current study suggests that CHIP confers resistance to apoptosis and acts as a potentiation factor in WJMSCs to provide protection from degenerative effects of diabetes.

## 1 INTRODUCTION

Diabetic patients exhibited an increased risk of heart failure.^1^, ^2^, ^3^ Lack of insulin, pregnancy, or insulin resistance may lead to the hyperglycemic condition.^4^ Hyperglycemia can induce apoptosis in many tissues and cells^5^, ^6^ that triggered the generation of reactive oxygen species (ROS), cardiac apoptosis, leading to the complication of diabetic cardiomyopathy.^7^, ^8^ Diabetic cardiomyopathy is a condition associated with abnormal ventricular function in diabetic patients in the absence of any other risk factors, such as hypertension and coronary atherosclerosis that occurs as a result of abnormal lipid and glucose metabolism resulting in the elevation of oxidative stress and other signaling cascades.^9^, ^10^, ^11^ Number of studies has shown that hyperglycemic conditions, such as diabetes induced apoptosis, senescence, and reduce the proliferation ability of mesenchymal stem cells.^12^, ^13^, ^14^ According to the previous study, diabetic condition limit survival of the transplanted stem cells by initiating apoptosis leading to cell death.^15^ Insulin, hypoglycemic agents, and dietary control are the currently available therapeutic strategies using worldwide for diabetes and its associated complications have limitations.^16^ Therefore, there is a need for cell‐based therapy to overcome this problem.

Wharton's jelly derived mesenchymal stem cells (WJMSCs) are the fibroblast‐like, highly homogenous population of cells that differ it from other stem cell sources.^17^, ^18^ The unique characteristics of WJMSCs are therapeutic potential, immune privilege, ease of isolation, and multidifferentiation potential.^19^ These properties make the WJMSCs as an ideal source for the treatment of many organs.^20^ The stem cells have the differentiation ability which can replace the dead cells, and release certain factors that trigger nearby cells in the microenvironment to accelerate the repair process.^21^ The effect of WJMSCs on diabetes has been widely investigated however, much less attention has been paid to elucidate whether WJMSCs have therapeutic potential against diabetes induced cardiac damages. Moreover, the reduction in the survival of the transplanted stem cells under hyperglycemic condition still remains as a concern for their clinical use.

Glucose intolerance is one of the phenomena often associated with diabetes particularly in the Type II diabetes. Conditions like glucose intolerance and obesity are often associated with upregulated levels of phosphatase and tensin homolog (PTEN).^22^ PTEN regulation is often associated with maintenance of survival mechanism and tissue homeostasis in various tissues, such as heart. Since maintenance of the survival and proliferation potential of stem cells are crucial criteria for a success transplantation therapy, PTEN knockdown is correlated with better stem cell function and repair.^23^ It is widely accepted that the level of PTEN should be tightly regulated as it involved in numerous cellular processes. NEDD4‐1, XIAP, and WWP2 are E3 ubiquitin ligase that maintains the level of PTEN via the ubiquitin‐proteasome system. Among them, NEDD4‐1 is the first identified E3 ligase that polyubiquitinates PTEN, which results in PTEN degradation.^24^, ^25^ PTEN acts as a tumor suppressor protein that negatively regulates the PI3K/Akt signaling pathway by converting PIP3 back to PIP2.^26^, ^27^ Previous study demonstrated that inhibition of PTEN reverses the hyperglycemic effects in mice.^28^ In another study, it was revealed that change in PTEN expression level regulates the muscle protein degradation in diabetic mice.^29^ Besides, there is not enough evidence about the increase or change in the localization of PTEN in NEDD4‐1 knockout cells, suggesting the involvement of other E3 ligases that may target PTEN for ubiquitylation.^26^, ^30^ Therefore in this study, we identify the PTEN regulating chaperone that help in controlling the PTEN expression levels in order to improve the functions of WJMSCs and maintain tissue homeostasis in diabetic conditions.

The Carboxyl terminus of Hsc70 interacting protein (CHIP) cDNA encodes a 34.5 kDa well‐conserved protein that has around 98% sequence similarity with the mouse and ∼60% similarity with the fruit fly.^31^ CHIP contains a conserved U‐box domain at their C terminus having E3 ligase activity and an N terminal tetratricopeptide repeat (TPR) domain responsible for interaction with Hsc/p70 and HSP90 clients.^32^, ^33^ An earlier study demonstrated the protective effect of CHIP against myocardial injury induced apoptosis and oxidative stress in the CHIP‐sufficient animal model.^34^ A recent study from our lab highlighted the protective effect of CHIP against doxorubicin induced cardiomyocyte death.^35^ In another study, we have envisaged that isoproterenol induced cytotoxicity attenuated by Tid‐1s through CHIP mediated Gαs degradation.^36^ However, to the best of our knowledge, it is not fully elucidated that whether CHIP could exert a protective effect against hyperglycemia‐induced apoptosis and oxidative stress under diabetic condition.

Research has shown that high glucose (HG)‐induced apoptosis and oxidative stress exerts a negative effect on stem cell function. However, the underlying mechanism that attenuates HG‐induced apoptosis and oxidative stress in diabetes remains elusive. In this study, we hypothesized that CHIP overexpressing WJMSCs may prevent HG‐induced apoptosis and oxidative stress by promoting ubiquitin‐mediated proteasomal degradation of PTEN, and might exert therapeutic effects against diabetes‐associated cardiac damage.

## 2 RESULTS

### 2.1 HG induces apoptosis and oxidative stress via activation of PTEN and the downstream signaling cascade in WJMSCs

**FIGURE 1:**
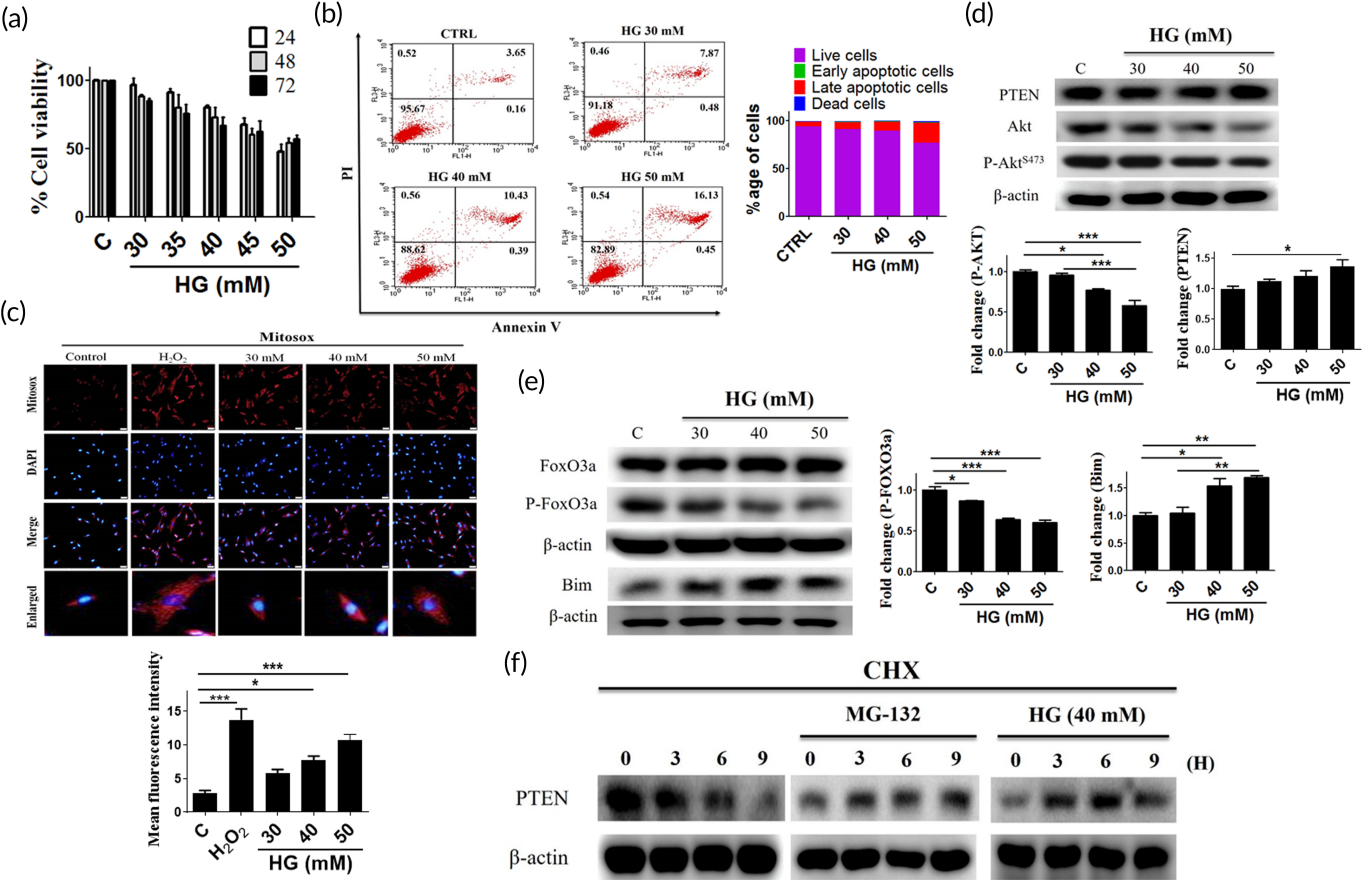
Effect of HG on PTEN‐mediated apoptosis and oxidative stress in WJMSCs. (a) WJMSCs seeded under varying concentration of HG for the indicated time points (24, 48, and 72 h) were harvested, and the cell viability was performed. (b, c) WJMSCs challenged with increasing concentrations of HG (30, 40, and 50 mM) for 24 h were incubated with the annexin V and PI and MitoSOX staining dye. The cell apoptosis and mitochondrial ROS were analyzed using flow cytometry and fluorescence microscopy. (d, e) WJMSCs were challenged with HG for 24 h, and the total cell lysate was harvested to analyze the expression of PTEN and downstream signaling cascade (AKT, p‐AKT, FOXO3a, p‐FOXO3a, and *bim*) via immunoblotting. (f) WJMSCs seeded in the presence of cycloheximide (CHX) were incubated with either MG‐132 (10 μM) or HG (40 mM) for the indicated time (0, 3, 6, 9 h) and thereafter immunoblotted with the anti‐PTEN antibody. β‐actin served as a loading control. The scale bar indicates 50 μm. Values shown are means ± SD. Quantification of the results are shown (*n* = 3). *p < 0.05, **p < 0.01, and ***p < 0.001 indicates the significant difference. HG, high glucose; PI, propidium iodide; PTEN, phosphatase and tensin homolog; ROS, reactive oxygen species; WJMSCs, Wharton's jelly derived mesenchymal stem cells

Previous studies have demonstrated that HG induces apoptosis and oxidative stress in various cell lines. Considering these, first, we assessed the effect of HG on cell viability. We observed that the cell viability was considerably reduced in a time‐ and dose‐dependent manner in WJMSCs after being challenged with HG (Figure 1(a)). Thereafter, we assessed whether HG can induce apoptosis in WJMSCs using flow cytometry and Western blot. Results indicated that increasing HG concentrations reduced the percentage of viable cells whereas the percentage of apoptotic cells (early, late) was significantly elevated in a dose‐dependent manner (Figure 1(b)). HG is the key regulator of mitochondrial ROS generation. Interestingly, immunofluorescence microscopy imaging showed increased ROS production with increasing HG concentrations (Figure 1(c)). These results indicate that HG can reduce cell viability and induce apoptosis and oxidative stress in a dose‐dependent manner in WJMSCs. In earlier studies, it was reported that HG‐induced oxidative stress and apoptosis are regulated via the FOXO3a pathway.^37^ Therefore, we evaluated the impact of HG on PTEN and the downstream signaling cascade in WJMSCs. Following HG induction, Western blot data demonstrated that PTEN expression increased with concomitant impairment in p‐AKT and elevation of FOXO3a and the downstream regulator (*Bim*) in a dose‐dependent manner (Figure 1(d),(e)). Further, it was found that PTEN levels were considerably reduced in a time‐dependent manner following cycloheximide (CHX) treatment; however, treatment with CHX in the presence of MG‐132 and HG conditions stabilized their expression level, indicating the inefficient degradation of PTEN (Figure 1(f)). The above data suggest that the PTEN/FOXO3a/*Bim* signaling pathway may be involved in HG‐induced apoptosis in WJMSCs.

### 2.2 CHIP overexpressed WJMSCs attenuate HG‐induced PTEN‐mediated apoptosis and oxidative stress

**FIGURE 2:**
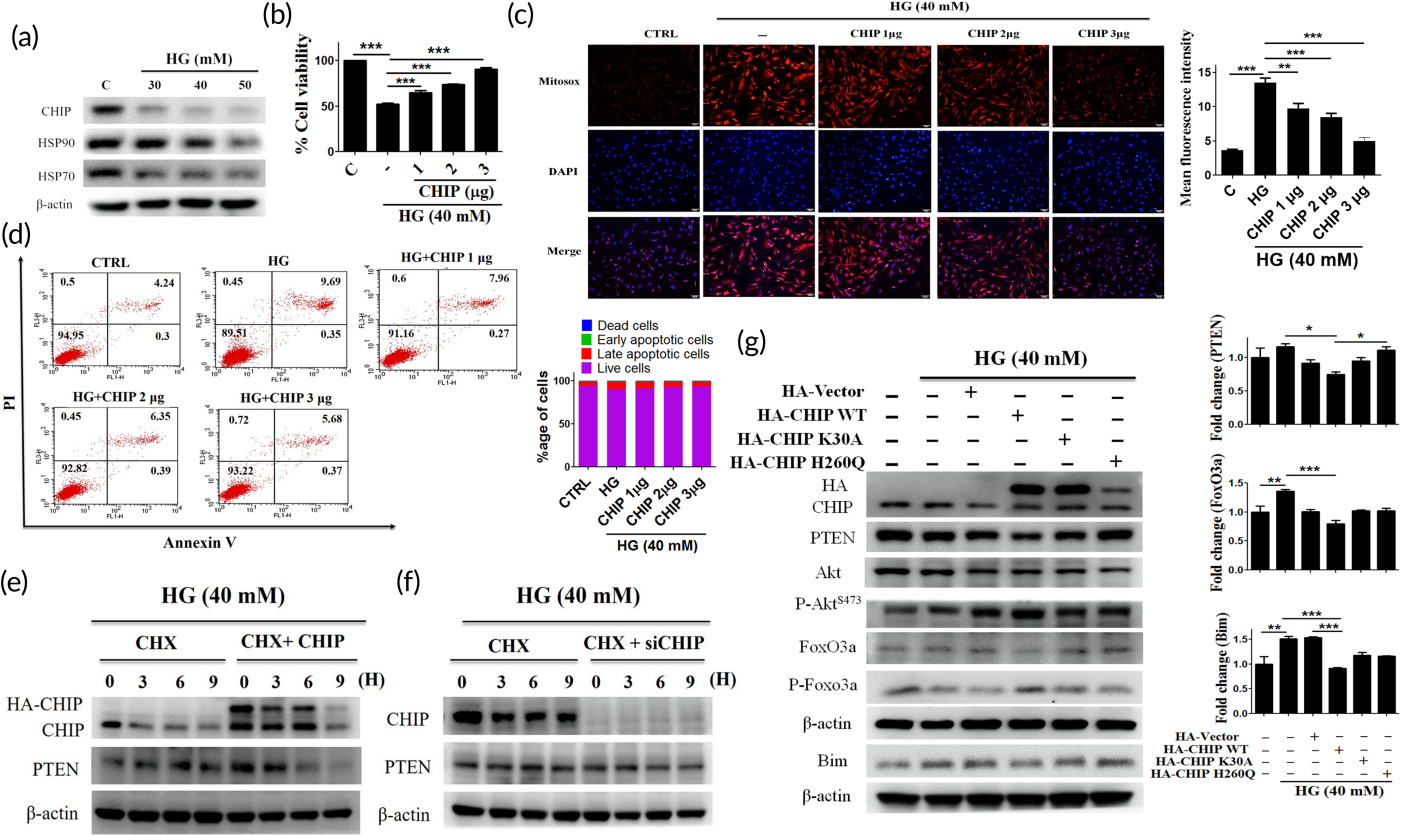
CHIP overexpressed WJMSCs attenuate hyperglycemia‐induced PTEN mediated apoptosis and oxidative stress. (a) WJMSCs were challenged with increasing concentrations of HG for 24 h, and subsequently the expression level of the chaperone system (HSP70, HSP90, and CHIP) was measured using Western blotting. (b) WJMSCs were transfected with varying increasing concentration of CHIP (1, 2, and 3 μg) followed by HG incubation for 24 h. The cell viability was detected. (C, D) WJMSCs were transfected with HA‐CHIP in the presence of HG (40 mM) for 24 h, and the mitochondrial ROS accumulation as well as apoptotic cell death were assessed using MitoSOX staining and flow cytometry. (e, f) WJMSCs were transfected with either HA‐CHIP (3 μg) or siCHIP (30 nM) for 24 h in the presence of CHX (50 μg/ml) for indicated time points were subjected to HG challenge for 24 h, and the protein expression was measured using Western blot analysis. (g) WJMSCs were transfected with pRK5‐HA‐vector (3 μg), pRK5‐HA‐CHIP (3 μg), pRK5‐HA‐K30A (3 μg), and pRK5‐HA‐H260Q (3 μg), followed by HG incubation for 24 h. Cell lysates were immunoblotted to analyze the expression of PTEN and the downstream signaling cascade. β‐actin act as a loading control. The scale bar indicates 100 μm. Values shown are means ± SD and quantification of the results shown as *n* = 3. *p < 0.05, **p < 0.01, and ***p < 0.001 indicates the significant difference. CHIP, carboxyl terminus of Hsc70 interacting protein; HG, high glucose; PTEN, phosphatase and tensin homolog; ROS, reactive oxygen species; WJMSCs, Wharton's jelly derived mesenchymal stem cells

Earlier studies demonstrated the protective role of CHIP against various stress conditions. Therefore, the molecular mechanism responsible for CHIP‐induced apoptosis and oxidative stress resistance was evaluated in WJMSCs. The expression of chaperones was analyzed after HG administration. Western blot analysis revealed that the expression of the chaperone system, including CHIP, HSP90, and HSP70, was reduced as compared to controls (Figure 2(a)). Besides, other E3 ligases, including NEDD4‐1, XIAP, and WWP2, that can ubiquitinate PTEN were analyzed using Western blotting and no observable difference was noticed in their expression levels under HG stress (Figure S2(a)). Further, cell viability was retained dose dependently in CHIP overexpressing cells after HG induction (Figure 2(b)). Moreover, it was found that enhanced CHIP expression attenuated PTEN expression level, rescued AKT levels, which were impaired under HG conditions, promoted the phosphorylation of FOXO3a, and inhibited the binding of FOXO3a with *Bim* in a dose‐dependent manner (Figure S2(a)). Next, we evaluated whether CHIP can attenuate HG‐induced apoptosis and mitochondrial ROS generation under HG conditions. Flow cytometry and MitoSOX staining ascertained that CHIP overexpression suppressed apoptosis and mitochondrial ROS dose dependently, as compared to HG (Figure 2(c),(d)). Furthermore, we evaluated the influence of CHIP overexpression and knockdown on endogenous PTEN levels under HG conditions, followed by cycloheximide treatment. The half‐life of PTEN was increased and decreased after transfection with siCHIP or CHIP, respectively (Figure 2(e),(f)). We further assessed whether CHIP has the potential to attenuate the expression of PTEN and inhibit its downstream signaling cascade under HG stress. For this, we employed various CHIP mutants, namely K30A (TPR domain mutant) and H260Q (U‐box domain mutant), together with wild‐type CHIP. Notably, it was found that wild‐type CHIP suppressed PTEN and FOXO3a expression, leading to the induction of p‐AKT and p‐FOXO3a protein expression levels. However, the vector and CHIP mutants (K30A and H260Q) did not exhibit any effects (Figure 2(g)). Collectively, this data demonstrate that CHIP suppressed HG‐induced PTEN expression, reduced its half‐life, and prevented apoptosis and oxidative stress; nevertheless, neither of the CHIP mutants (H260Q and K30A) were able to attenuate PTEN in WJMSCs. Multiple prediction methods were used to identify binding sites based on structure. The docking method with GOLD 3.0.1 predicted 10 confirmations and binding scores. Our analysis revealed that the N‐terminal of the CHIP domain has a high tendency for the N‐terminal of the PTEN domain, and are potentially involved in binding. The total clusters of docking conformations with the docked PTEN showed positive binding energies. Among all docking conformations, the best predicted binding free energy is −125.34 kJ/mol to PTEN (Figure S2(b)).

### 2.3 CHIP regulates PTEN and its downstream signaling cascade under HG conditions in WJMSCs

**FIGURE 3:**
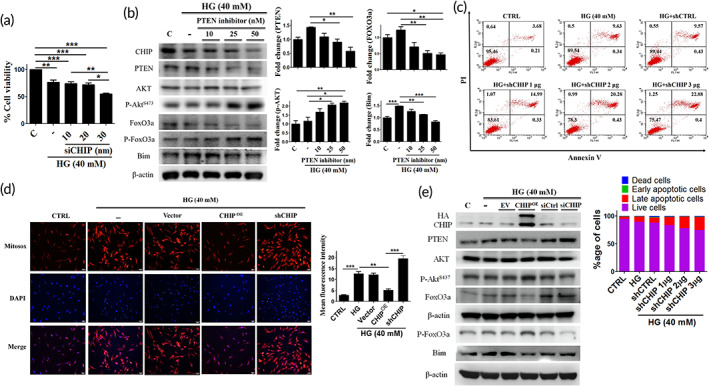
CHIP regulates PTEN and its downstream signaling mediators under HG conditions. (a) Cells were transfected with increasing amounts of siCHIP (10, 20, 30 nM), and cell viability was determined after challenged with HG for 24 h. (b) WJMSCs incubated with varying increasing concentration of PTEN inhibitor (10, 25, 50 nM) were subjected to HG for 24 h, and the total cellular extract was analyzed using Western blot analysis. (c, d) Cells transfected with HA‐vector (3 μg), HA‐CHIP (3 μg), and shCHIP (3 μg) were challenged with HG for 24 h, and flow cytometry as well as MitoSOX staining was performed to analyze the apoptosis rate and mitochondrial ROS production. (e) WJMSCs were transfected with empty vector (EV) (3 μg), HA‐CHIP (3 μg), sicontrol (siCtrl) (30 nM), or siCHIP (30 nM) in the presence of HG for 24 h. Whole cell lysate was analyzed via immunoblotting. β‐actin served as a loading control. The scale bar indicates 100 μm. Values shown are mean ± SD. Quantification of the results are shown (*n* = 3). *p < 0.05, **p < 0.01, and ***p < 0.001 indicates the significant difference. CHIP, carboxyl terminus of Hsc70 interacting protein; HG, high glucose; PTEN, phosphatase and tensin homolog; ROS, reactive oxygen species; WJMSCs, Wharton's jelly derived mesenchymal stem cells

CHIP can downregulate PTEN, thereby inhibiting HG‐induced apoptosis and oxidative stress. Therefore, we evaluated the impact of CHIP knockdown on cell viability and PTEN and its downstream signaling cascade under HG conditions. The cell viability was reduced dose dependently upon CHIP depletion (Figure 3(a)), and showed a significant increase in PTEN, FOXO3a, and *Bim* protein expression levels in a dose‐dependent manner, as compared to control and HG condition alone. However, the expression of phosphorylated AKT and FOXO3a was downregulated in the presence of siCHIP (Figure S3(a)). Next, we determined the effect of PTEN inhibition on the downstream signaling pathway. The protein expression of p‐AKT appeared increased, while the FOXO3a and *Bim* interaction was inhibited in a dose‐dependent manner after HG exposure (Figure 3(b)). We further validated the effects of CHIP on apoptosis and oxidative stress under HG conditions. Flow cytometry and MitoSOX staining revealed that CHIP overexpression suppressed HG‐induced apoptosis and ROS generation, and the effects were reversed upon CHIP knockdown (Figure 3(c),(d) and S3(b)). CHIP knockdown elevated the expression of PTEN and the downstream signaling mediators, which indicates that CHIP regulates the PTEN/FOXO3a/*Bim* signaling pathway (Figure 3(e)). Altogether, these data suggest that CHIP regulates PTEN and the downstream signaling cascade under HG stress in WJMSCs.

### 2.4 CHIP targets PTEN for ubiquitin‐mediated proteasomal degradation cooperated by HSP70 under HG conditions

**FIGURE 4:**
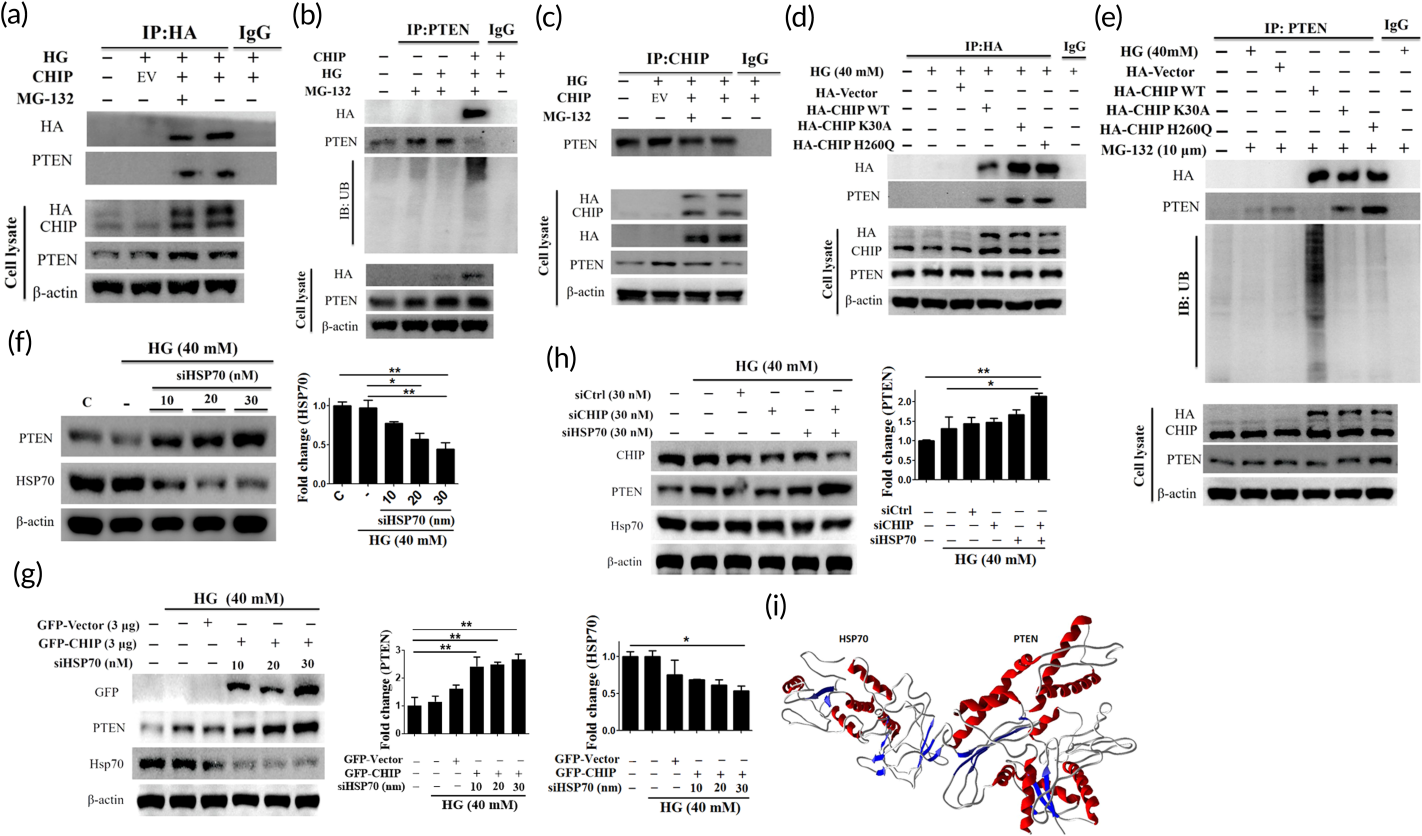
CHIP targets HG induced‐PTEN for ubiquitin‐mediated proteasomal degradation cooperated by HSP70 under HG conditions. (a–c) Cells transfected with HA‐vector or HA‐CHIP in the presence and absence of MG‐132 for 6 h were subjected to HG challenge for 24 h. Whole cell lysate was immunoprecipitated with the anti‐HA, anti‐CHIP, and anti‐PTEN antibody, and subsequently immunoblotted with the primary antibodies, including anti‐HA, anti‐PTEN, and anti‐ubiquitin. (d, e) Cells transfected with HA‐vector, HA‐CHIP, and CHIP mutants (K30A, an H260Q) were treated with or without MG‐132 for 6 h in the presence of HG for 24 h. Whole cell lysate was immunoprecipitated with the anti‐HA and anti‐PTEN antibody followed by immunoblotting with the anti‐HA, anti‐PTEN, and anti‐ubiquitin antibody. (f) Cells were transfected with increasing concentrations of siHSP70 (10, 20, 30 nM) after challenged with HG for 24 h, and the expression level of PTEN and HSP70 was measured employing Western blot analysis. (g) Following cotransfection of GFP‐vector or GFP‐CHIP with increasing concentration of siHSP70 in WJMSCs were challenged with HG for 24 h, and the protein expression was measured via immunoblotting. (h) WJMSCs transfected with sicontrol, CHIP siRNA, or siHSP70 were subjected to HG challenge for 24 h, and the total cell extract was immunoblotted with CHIP, PTEN, and HSP70. β‐actin served as a loading control. (i) Docking studies demonstrating the molecular interaction of HSP70 with PTEN forming a heteromer complex (HSP70 and PTEN shown in quaternary structure with helices and sheets in complex). Values shown are mean ± SD. Quantification of the results are shown (*n* = 3). *p < 0.05, **p < 0.01, and ***p < 0.001 indicates the significant difference. CHIP, carboxyl terminus of Hsc70 interacting protein; HG, high glucose; PTEN, phosphatase and tensin homolog; WJMSCs, Wharton's jelly derived mesenchymal stem cells

Above data indicate that CHIP overexpression attenuates HG‐induced PTEN, and this effect was reversed upon CHIP knockdown. Considering these, we evaluated whether CHIP promotes the ubiquitin‐mediated proteasomal degradation of PTEN. Co‐immunoprecipitation (Co‐IP) data showed that CHIP directly interacts with PTEN and promotes its ubiquitination (Figure 4(a)–(c)). These results indicate that CHIP has the potential to interact with and promote ubiquitin‐mediated proteasomal degradation of PTEN. Further, co‐IP was performed to analyze the binding ability and E3 ligase activity of CHIP. The co‐IP data revealed that both CHIP mutants have the binding ability, but neither have the potential to ubiquitinate PTEN, which indicates that both domains (K30A and H260Q) are responsible for ubiquitin‐mediated proteasomal degradation of PTEN, and the possible involvement of chaperones, as the K30A mutant loses its ability to interact with HSP70 and 90 (Figure 4(d),(e)). It is well known that CHIP regulates various proteins presented by the HSP70. Therefore, we evaluated the influence of HSP70 in CHIP‐mediated PTEN degradation. We found that HSP70 inhibition led to a dose‐dependent increase in PTEN protein expression (Figure 4(f)). Further, HSP70 inhibition blocks the loss of PTEN in the presence of CHIP and/or siCHIP after HG exposure (Figure 4(g),(h)). Besides, the molecular interaction of HSP70 and PTEN was verified using In Silico analysis (Figure 4(i)). These data demonstrate that HSP70 co‐operates with CHIP to promote PTEN degradation under HG conditions. Collectively, these data suggest that CHIP promotes ubiquitin‐mediated proteasomal degradation of PTEN might be co‐operated by HSP70 under HG conditions in WJMSCs.

### 2.5 CHIP regulates the binding of FOXO3a with the *Bim* promoter region

**FIGURE 5:**
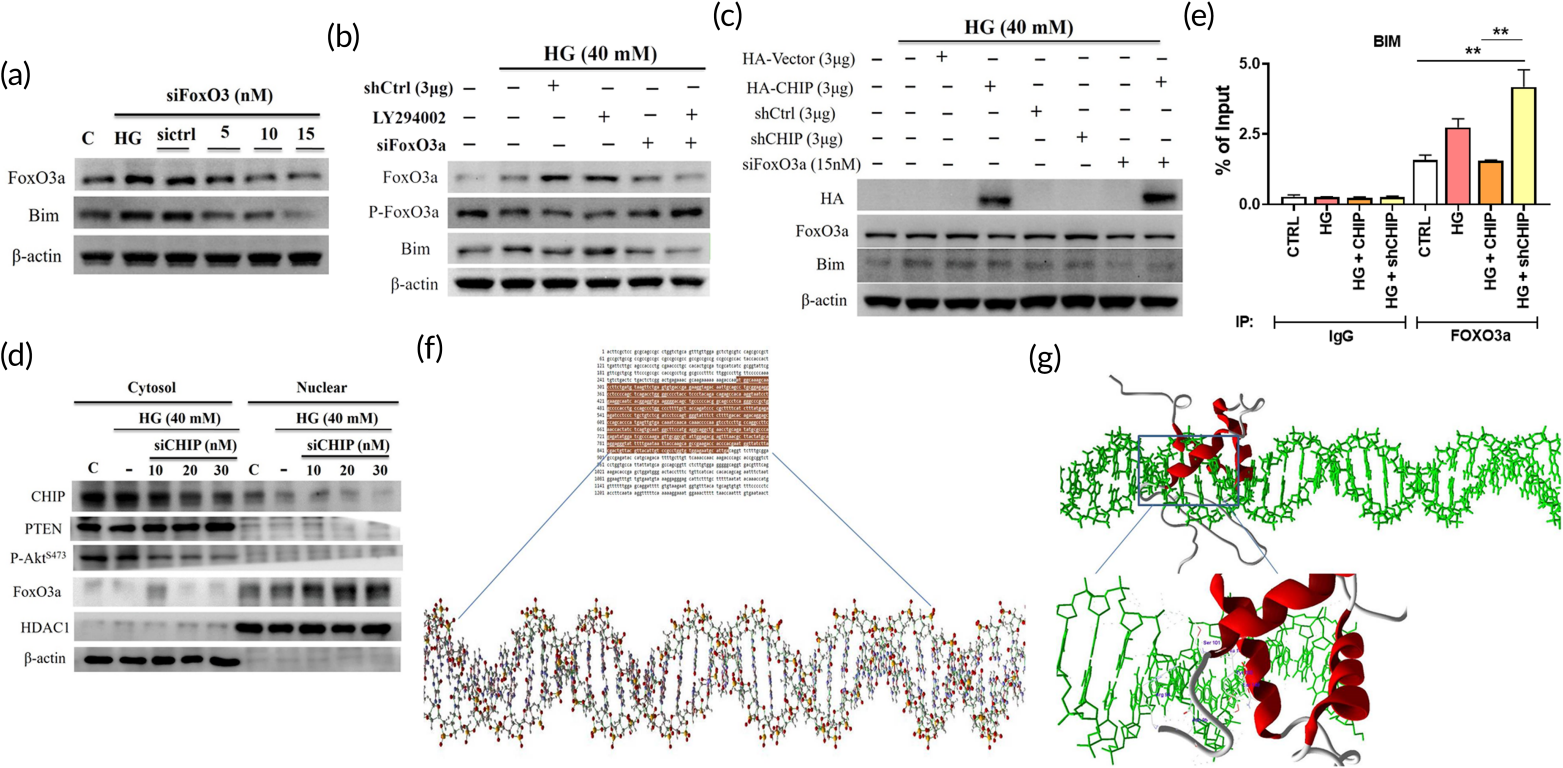
CHIP regulates the binding of FOXO3a with the *Bim* promoter region. (a) WJMSCs transfected with increasing amounts of siFOXO3a were challenged with HG stress, and the expression levels of FOXO3a and *Bim* were assessed by immunoblotting. (b) Cells transfected with shcontrol or siFOXO3a in the presence and absence of LY294002 (PI3K inhibitor) were subjected to HG for 24 h, and the activation of FOXO3a, p‐FOXO3a, and *Bim* was analyzed using Western blot assay. (c) WJMSCs transfected with HA‐vector, HA‐CHIP, shcontrol, shCHIP, or siFOXO3a were incubated with HG for 24 h followed by immunoblotting to analyze the HA, FoxO3a, and *Bim* levels. (d) WJMSCs were transfected with varying concentration of siCHIP (10, 20, 30 nM) in the presence of HG for 24 h. Thereafter, the extracted cell lysates using cytoplasmic and nuclear fractionation kit were immunoblotted. (e) WJMSCs transfected with HA‐CHIP or shCHIP plasmids were challenged with HG for 24 h and ChIP assay was performed to evaluate the FOXO3a interaction with the *Bim* promoter region. (f) Prediction of *Bim* promoter region using NCBI database (brown color indicates *Bim* promoter region) and converted to three‐dimensional structure for molecular interaction with FOXO3a. (g) Docking studies illustrating the molecular interaction between FOXO3a and the *Bim* promoter region (Bim region is shown in green color and FOXO3a shown in red color). *p < 0.05, **p < 0.01, and ***p < 0.001 shows the significance. CHIP, carboxyl terminus of Hsc70 interacting protein; HG, high glucose; WJMSCs, Wharton's jelly derived mesenchymal stem cells

FOXO3a, a vital transcription factor, can bind to various promoters, including *Bim*. Therefore, we assessed whether FOXO3a knockdown can influence the expression of *bim* under HG conditions. From the immunoblot assay, we observed that *Bim* was downregulated dose dependently upon FOXO3a knockdown under HG conditions (Figure 5(a)). Thereafter, we silenced FOXO3a together with AKT, and observed that AKT inhibition hindered elevation of the proapoptotic protein *Bim* (Figure 5(b)). We performed Western blotting to further ascertain the effect of CHIP on FOXO3a and its downstream promoter. Western blot analysis revealed that CHIP overexpression inhibited FOXO3a expression and the downstream *bim* promoter; whereas, CHIP knockdown reversed this effect, indicating that CHIP regulates the binding of FOXO3a with the *Bim* promoter region via activation of AKT (Figure 5(c)). Next, we determined whether CHIP regulates PTEN and FOXO3a protein expression in WJMSCs. As shown in Figure 5(d), HG‐induced cytoplasmic PTEN and nuclear FOXO3a protein levels were further increased by silencing CHIP in a dose‐dependent manner (Figure 5(d)). Furthermore, chromatin immunoprecipitation (ChIP) assay was performed to analyze whether CHIP overexpression regulates the binding of FOXO3a with the *Bim* promoter region. It was revealed that HG increased the binding ability of FOXO3a with the *Bim* promoter fragment was reduced following CHIP overexpression; whereas, CHIP knockdown further increased their interaction (Figure 5(e)). The active site of FOXO3a was predicted using the CASTp server, and includes amino acids TRP157, LEU160, LEU165, ARG168, CYS190, VAL190, and PRO192 (Figure S5(b)). FOXO3a binds to the *Bim* promoter with high affinity and induces apoptosis. The *Bim* promoter region was selected from the NCBI database and drawn using Avogadro software (Figure 5(f)). Docking studies were performed to gain insight into the binding conformation of FOXO3a with the *Bim* promoter region. All docking calculations were carried out using GOLD and the files generated were analyzed for their binding conformations. Among the active residues, ARG94, ASP95, SER101, TYR102, and SER149 play an important role in forming hydrogen bonds with the *Bim* promoter region (Figure 5(g)).

### 2.6 Coculture of CHIP overexpressing WJMSCs with cardiac cells ameliorates HG‐induced cardiac apoptosis and oxidative stress

**FIGURE 6:**
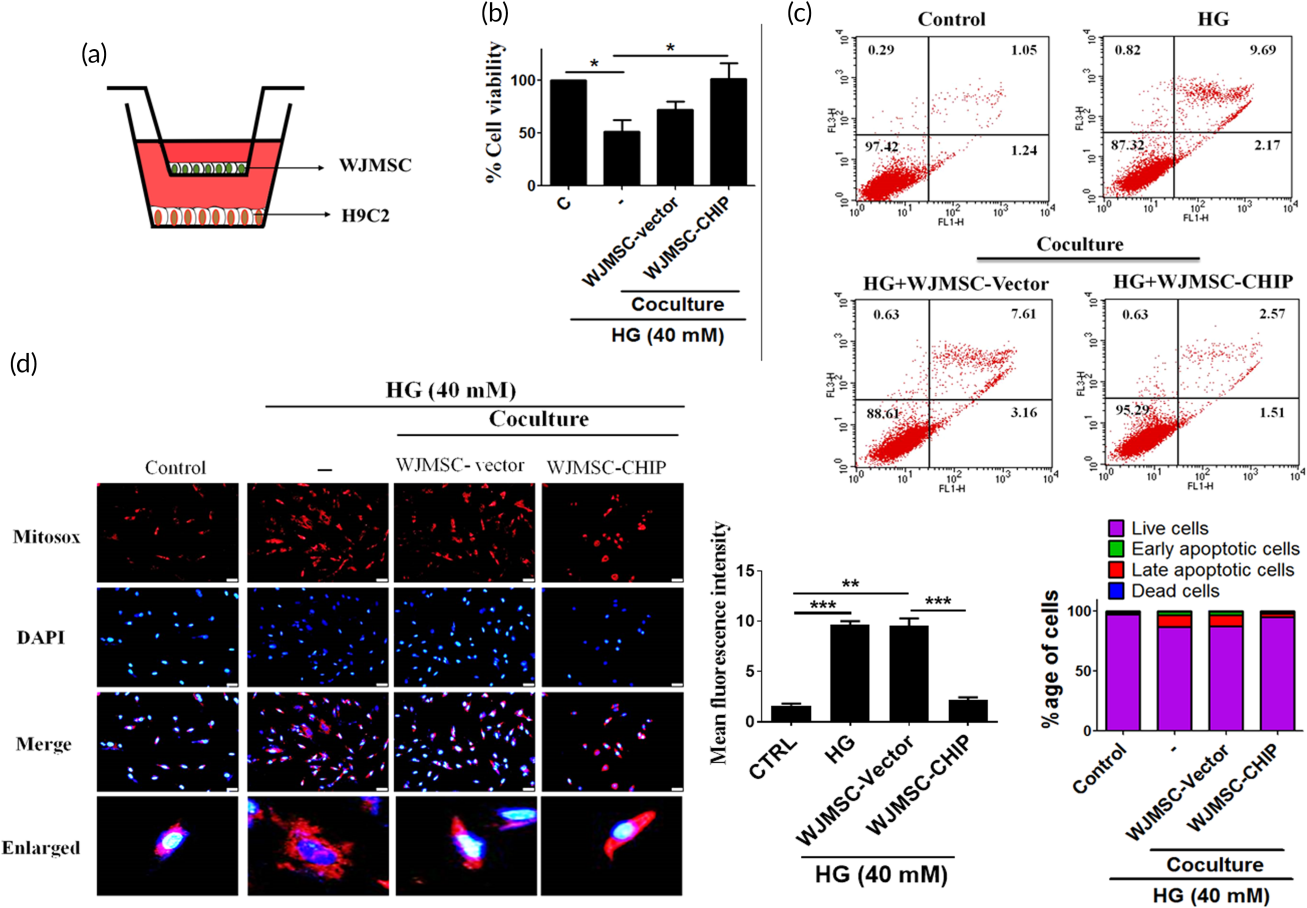
Coculturing CHIP overexpressed WJMSCs with cardiac cells rescued HG‐induced cardiac apoptosis and oxidative stress. (a) Schematic representation of in vitro coculturing WJMSCs with H9c2 cardiomyoblasts. (b) WJMSCs transfected with HA‐vector or HA‐CHIP in the presence of HG were cocultured with cardiomyoblasts followed by incubation with MTT reagent to assess the cell viability. (c, d) Embryo derived cardiac cells were cocultured with WJMSCs alone, WJMSCs transfected with HA‐vector, or HA‐CHIP in the presence of HG stress for 24 h. Flow cytometry and MitoSOX staining were performed to estimate the apoptotic cell death and mitochondrial oxidative stress generation. The scale bar indicates 50 μm. β‐actin acts as a loading control. Values shown are mean ± SD. Quantification of the results are shown (*n* = 3). *p < 0.05, **p < 0.01, and ***p < 0.001 represents the significance. CHIP, carboxyl terminus of Hsc70 interacting protein; HG, high glucose; WJMSCs, Wharton's jelly derived mesenchymal stem cells

Figure 6(a) indicates a schematic illustration of the in vitro coculturing system, in which WJMSCs were cultured in the upper chamber, with cardiac cells in the lower chamber (Figure 6(a)). We determined whether CHIP overexpressed WJMSCs could rescue cell viability. The data indicates that coculturing CHIP overexpressing WJMSCs significantly retained the cell viability in H9c2 cells as compared to HG and WJMSCs alone (Figure 6(b)). Next, we assessed whether coculturing of WJMSCs with H9c2 could rescue HG‐induced cardiac apoptosis and oxidative stress. Flow cytometry and MitoSOX staining showed that HG considerably induces apoptotic cells and oxidative stress. However, coculturing with WJMSCs, especially CHIP overexpressing WJMSCs, rescued HG‐induced injuries in embryo derived cardiomyoblasts (Figure 6(c),(d)). All these data demonstrate that CHIP overexpressed WJMSCs exert protective effects against HG‐induced cardiac apoptosis and oxidative stress.

### 2.7 CHIP overexpressed WJMSCs ameliorated hyperglycemia‐induced cardiac damage in streptozotocin (STZ)‐induced diabetic rats

**FIGURE 7:**
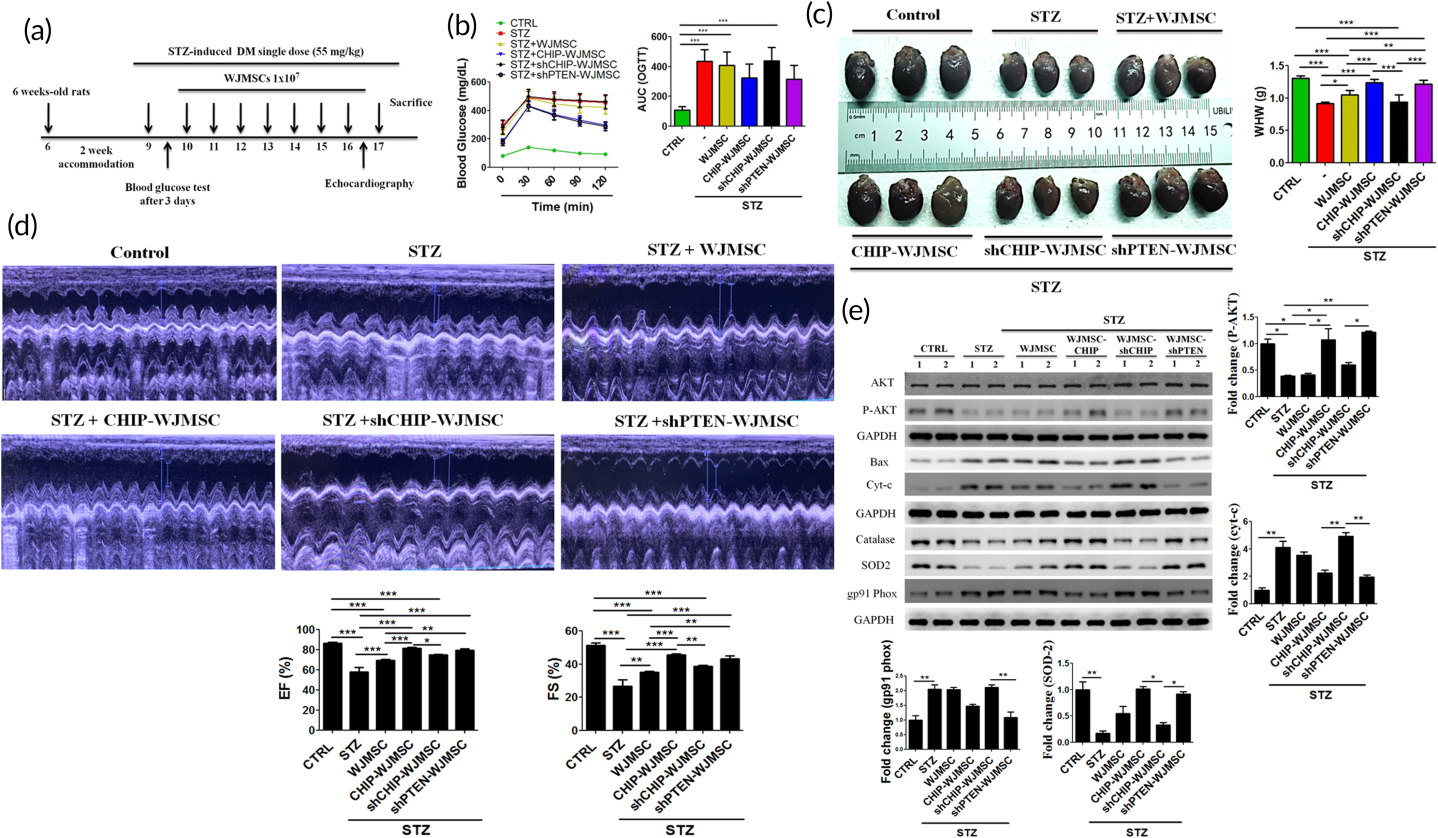
CHIP overexpressed WJMSCs rescued hyperglycemic effects under diabetic conditions. (a) Schematic illustration of STZ‐induced diabetes, and WJMSCs administration expressing different plasmids, including GFP‐CHIP, shCHIP, and shPTEN. (b) The oral glucose tolerance test (OGTT) was performed after 6 weeks treatment for the indicated time points (0, 30, 60, 90, and 120 min) in various experimental groups, including control, STZ‐induced diabetes (STZ), STZ‐induced diabetes administered with WJMSCs alone (STZ + WJMSCs), STZ‐induced diabetes injected with CHIP overexpressed WJMSCs (STZ + CHIP‐WJMSCs), STZ‐induced diabetes transplanted with CHIP knockdown WJMSCs (STZ + shCHIP‐WJMSCs), and STZ‐induced diabetic rats infused with PTEN knockdown WJMSCs (STZ + shPTEN‐WJMSCs) after the rats were fasted for 14 h. (c) Morphological assessment of cardiac tissues in different experimental groups. (d) Echocardiographic evaluation of cardiac function in different experimental groups (control, STZ, STZ + WJMSCs, STZ + CHIP‐WJMSCs, STZ + shCHIP‐WJMSCs, and STZ + shPTEN‐WJMSCs). (e) Total cell lysate from the left ventricle was quantified and measured using Western blot. Protein expression levels of the apoptosis (p‐AKT, Bax, and Cyt‐c) and oxidative stress markers (catalase, SOD2, and gp91^PHOX^) were assessed. GAPDH act as a loading control. Values shown are mean ± SD. Quantification of the results are shown (*n* = 3). *p < 0.05, **p < 0.01, and ***p < 0.001 shows the significance. CHIP, carboxyl terminus of Hsc70 interacting protein; GAPDH, Glyceraldehyde‐3‐phosphate dehydrogenase; PTEN, phosphatase and tensin homolog; STZ, streptozotocin; WJMSCs, Wharton's jelly derived mesenchymal stem cells

Finally, we assessed whether CHIP overexpressed WJMSCs rescued hyperglycemia‐induced cardiac injury in vivo. Before STZ induction, all rats exhibited normal body weight and blood glucose levels. First, diabetes was induced using STZ in male Sprague‐Dawley (SD) rats, and after 4 days animals received WJMSCs alone or WJMSCs carrying lentiviral plasmids, including GFP‐CHIP, shCHIP, and shPTEN (Figure 7(a)). Oral glucose tolerance test (OGTT) was performed to evaluate the antihyperglycemic effects in all experimental groups. STZ‐induced diabetes, WJMSCs alone, and shCHIP‐WJSMCs groups exhibited markedly increased blood glucose levels and area under the curve (AUC) obtained from OGTT, as compared to the control group. However, CHIP overexpressed WJMSCs, and shPTEN expressing WJMSCs attenuated the blood glucose levels and AUC as well (Figure 7(b)). It was also noticed that no signs of tumorigenicity were observed during tissue sectioning of experimental animals after injecting WJMSCs carrying CHIP or shPTEN lentiviral plasmids. Moreover, it was observed that whole heart weight (WHW) and left ventricle weight (LVW) were markedly reduced in STZ‐induced diabetes, WJMSCs alone, and WJMSCs carrying shCHIP groups. However, CHIP overexpression and PTEN knockdown in WJMSCs significantly rescued the WHW and LVW weight in the experimental rats (Figure 7(c)). Besides, LVW/WHW, WHW/tibia length (TL), and LVW/TL exhibited obvious reduction compared to the control group. Interestingly, transplantation of CHIP overexpressed and PTEN knockdown WJMSCs rescued the above‐mentioned parameters (Table 1). Echocardiography was performed to evaluate the cardiac function in experimental rats. The cardiac parameters related to cardiac function were reduced in STZ‐induced diabetes, WJMSCs alone, and shCHIP containing WJMSCs as compared to the control group. The ejection fraction (EF) and fractional shortening (FS) were obviously reduced in STZ‐induced diabetes, WJMSCs, and shCHIP expressing WJMSCs groups. Moreover, other parameters, such as interventricular septum at diastole, internal dimension at diastole of the left ventricle, end‐diastolic volume, stroke volume, and left ventricular diameter mass (LVD), were also lowered in STZ‐induced diabetes, and shCHIP carrying WJMSCs groups. Interestingly, infusion of CHIP overexpressed and PTEN silenced WJMSCs significantly improved the above‐mentioned parameters back to normal levels (Figure 7(d), Table 2). Further, Western blot analysis validated the apoptosis and oxidative stress markers with abnormal protein expression in the left ventricle of STZ, WJMSCs, and shCHIP‐WJMSCs reverted to normal levels in the groups with enhanced CHIP and PTEN‐silenced WJMSCs in the presence of STZ (Figure 7(e)). These data indicate that CHIP overexpressing WJMSCs provides protective effects against hyperglycemic induced cardiac injuries under diabetic conditions.

**TABLE 1:** Morphological assessment of the experimental rats

	Control (*n* = 9)	STZ (*n* = 9)	STZ + WJMSC (*n* = 9)	STZ + CHIP‐WJMSC (*n* = 9)	STZ + shCHIP‐WJMSC (*n* = 9)	STZ + shPTEN‐WJMSC (*n* = 9)
*Before STZ injection*						
BW (g)	245.4 ± 10.1	242.2 ± 12.6	246.5 ± 13.1	240.1 ± 11	238.9 ± 11.4	236.7 ± 5.5
BS (mg/dl)	122.5 ± 2.2	124.4 ± 3.6	120 ± 6.4	122.5 ± 7	120.8 ± 6.9	119.7 ± 5.3
*After STZ injection*						
BW (g)	366.7 ± 9.65	245 ± 12.8***	267 ± 12.2***	302.1 ± 11.9**#	238.1 ± 11.1***	295 ± 8.6**
BS (mg/dl)	119.4 ± 4.6	504 ± 43.6***	460.4 ± 42.8***	415.5 ± 35.8***#	513.1 ± 43.5***	403 ± 32.3***##
WHW (g)	1.34 ± 0.038	0.98 ± 0.019***	1.04 ± 0.074***#	1.23 ± 0.053###	0.93 ± 0.11***	1.22 ± 0.05###
LVW (g)	1.11 ± 0.03	0.67 ± 0.01***	0.78 ± 0.02***###	0.95 ± 0.02***###	0.66 ± 0.018***	0.955 ± 0.052***###
LVW/WHW	0.85 ± 0.028	0.73 ± 0.015*	0.74 ± 0.044*	0.77 ± 0.034	0.72 ± 0.085**	0.78 ± 0.055
TL (mm)	43.05 ± 0.56	42.18 ± 0.46	42.2 ± 0.39	42.8 ± 0.54	42 ± 0.38	42.9 ± 0.29
WHW/TL (g/mm)	0.03 ± 0.00078	0.021 ± 0.00053***	0.022 ± 0.00157***#	0.029 ± 0.00118###	0.02 ± 0.00226***	0.028 ± 0.00126###
LVW/TL (g/mm)	0.025 ± 0.00062	0.016 ± 0.00044***	0.018 ± 0.00067***###	0.022 ± 0.00058***###	0.015 ± 0.00036***	0.022 ± 0.00117***###

*Note*: Values are presented as mean ± SD of male SD rats containing control, STZ‐induced diabetes, STZ‐induced diabetes administered with WJMSCs, STZ‐induced diabetes injected with WJMSCs expressing lentiviral GFP‐CHIP, STZ‐induced diabetes transplanted with WJMSCs carrying lentiviral shCHIP and shPTEN plasmids. Values presented are mean ± SD. *p < 0.05, **p < 0.01, and ***p < 0.001 are compared to control group; ^#^ *p* < 0.05, ^##^ *p* < 0.01, and ^###^ *p* < 0.001 represents significance compared to STZ group.

Abbreviations: BS, blood sugar; BW, body weight; CHIP, carboxyl terminus of Hsc70 interacting protein; LVW, left ventricular weight; STZ, streptozotocin; TL, tibia length; WHW, whole heart weight; WJMSC, Wharton's jelly derived mesenchymal stem cell.

**TABLE 2:** Echocardiographic assessment of the cardiac function

	Control (*n* = 9)	STZ (*n* = 9)	STZ + WJMSC (*n* = 9)	STZ + CHIP‐WJMSC (*n* = 9)	STZ + shCHIP‐WJMSC (*n* = 9)	STZ + shPTEN‐WJMSC (*n* = 9)
IVSd (mm)	1.8 ± 0.13	1.4 ± 0.12*	1.55 ± 0.07	2 ± 0.07###	1.65 ± 0.3	1.7 ± 0.007#
LVIDd (mm)	8.35 ± 0.07	6.65 ± 0.35*	8.2 ± 1.2#	8.5 ± 0.8#	7.05 ± 0.7	8.55 ± 0.7#
LVIDs (mm)	4.1 ± 0.14	4.85 ± 0.6	5.5 ± 0.8**	4.5 ± 0.5	4.3 ± 0.4	4.85 ± 0.2
EDV (Teich) (ml)	1.2 ± 0.02	0.67 ± 0.09*	0.9 ± 0.6#	1.2 ± 0.3#	0.8 ± 0.2	1.3 ± 0.3#
ESV (Teich) (ml)	0.17 ± 0.01	0.28 ± 0.1	0.41 ± 0.17**	0.22 ± 0.07	0.2 ± 0.05	0.27 ± 0.03
EF (Teich) (ml)	86.71 ± 1.5	58 ± 8.68***	69.78 ± 1.01***###	81.79 ± 1.17###	74.94 ± 0.75***##	79.48 ± 2.21*###
%FS	51.37 ± 1.94	26.87 ± 6.57***	35.01 ± 1.06***##	45.62 ± 1.04*###	38.87 ± 0.81***##	43.4 ± 2.33**###
SV (Teich) (ml)	1.1 ± 0.04	0.38 ± 0.01**	0.75 ± 0.41#	0.99 ± 0.26##	0.6 ± 0.18*	1.08 ± 0.3##
LVd Mass (ASE) (g)	1.61 ± 0.01	1.27 ± 0.05***	1.33 ± 0.14***	1.73 ± 0.08###	1.26 ± 0.007***	1.5 ± 0.002##

Abbreviations: ASE, american society of echocardiography; EDV, end‐diastolic volume; EF, ejection fraction; ESV, end‐systolic volume; FS, fractional shortening; IVSd, interventricular septal thickness at diastole; LVD, left ventricular diameter; LVIDd, left ventricle inner dimension in diastole; LVIDs, left ventricle inner dimension in systole; STZ, streptozotocin; SV, stroke volume; WJMSC, Wharton's jelly derived mesenchymal stem cell.

### 2.8 CHIP overexpressing WJMSCs ameliorated hyperglycemia‐induced cardiac injuries in diabetic animals

**FIGURE 8:**
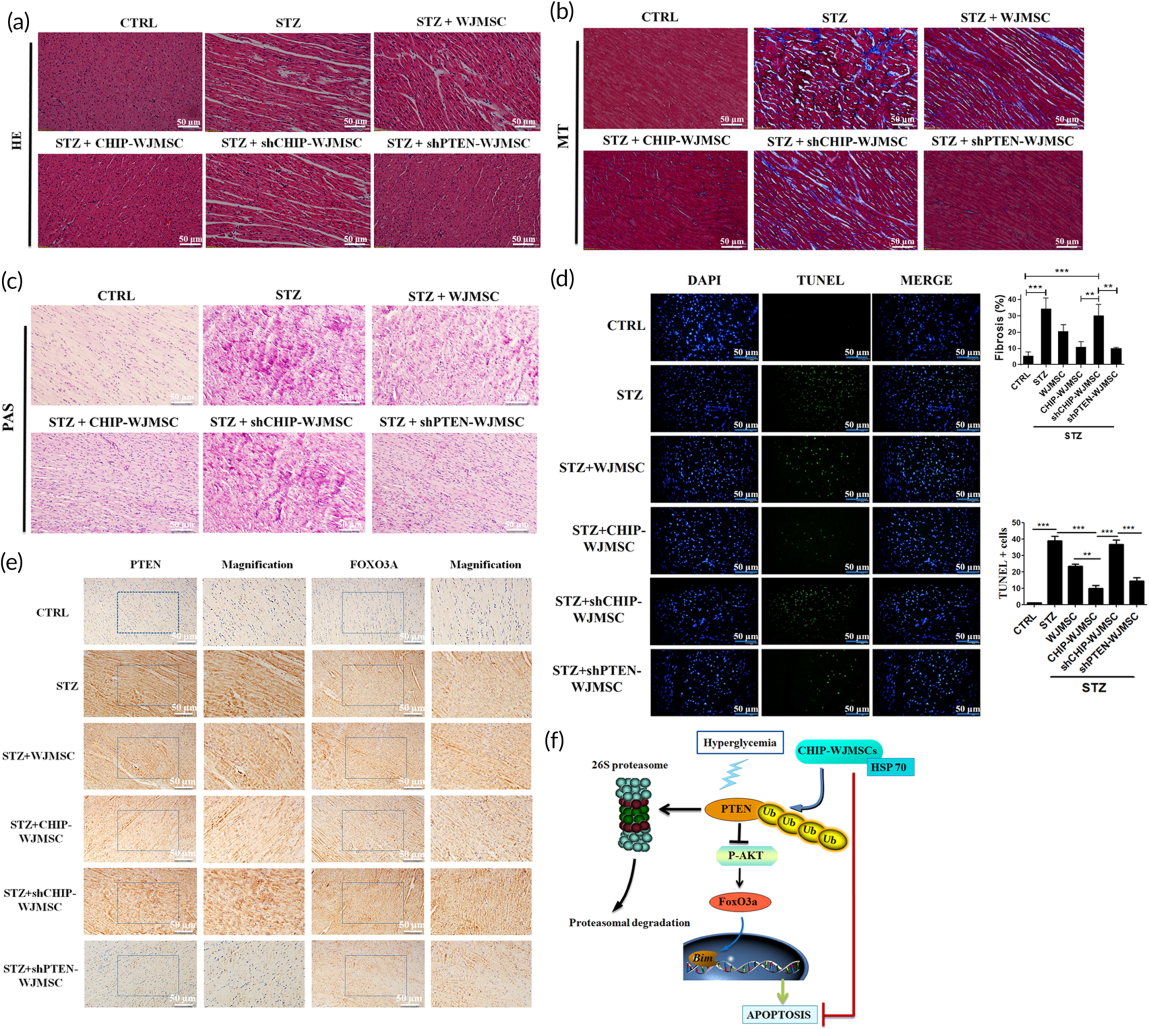
CHIP overexpressing WJMSCs ameliorated hyperglycemia‐induced cardiac injuries in diabetic animals. (a–c) Hematoxylin‐eosin (HE), Masson's trichrome (MT), and Periodic acid‐Schiff staining (PAS) were performed to evaluate cardiac morphology, fibrosis, and glycogen accumulation in different experimental groups. (d) Cardiomyocyte apoptosis was assessed using the TUNEL assay. (e) Immunohistochemistry assay (IHC) was performed to evaluate cardiac expression of PTEN and FOXO3a in different experimental groups. (f) Schematic illustration of CHIP‐mediated PTEN degradation under hyperglycemic conditions showed resistance to apoptosis. The scale bar indicates 50 μm. Values shown are mean ± SD. Quantification of the results are shown (*n* = 3). *p < 0.05, **p < 0.01, and ***p < 0.001 indicates the significant difference. CHIP, carboxyl terminus of Hsc70 interacting protein; PTEN, phosphatase and tensin homolog; TUNEL, Terminal deoxynucleotidyl transferase dUTP nick end labeling; WJMSCs, Wharton's jelly derived mesenchymal stem cells

Furthermore, hematoxylin eosin, Masson's trichrome, and periodic acid Schiff staining were performed to assess cardiac morphology in experimental animals. Results indicated that increased interstitial spaces, collagen representing fibrosis, and glycogen accumulation were observed in STZ, WJMSCs, and shCHIP‐WJMSCs groups, but the cardiac damage induced in these groups were rescued after infusion of CHIP overexpressed and PTEN knockdown WJMSCs (Figure 8(a)–(c)). Terminal deoxynucleotidyl transferase dUTP nick end labeling (TUNEL) positive cardiac cells induced by STZ‐induced diabetes, WJMSCs, and WJMSCs administered shCHIP were strongly reduced in groups injected with WJMSCs expressing CHIP and shPTEN plasmids (Figure 8(d)). Moreover, immunohistochemical imaging ascertained that the expression levels of PTEN and FOXO3a were elevated in groups with STZ, WJMSCs alone, and WJMSCs infused with shCHIP, as compared to controls. In contrast, transplantation of WJMSCs expressing CHIP and shPTEN reduced their expression level (Figure 8(e)). These results indicate that CHIP exerts protective effects against hyperglycemia‐induced cardiac injury in STZ‐induced diabetic rats by reducing PTEN stability in WJMSCs. Collectively, the present study suggests that CHIP targets PTEN for ubiquitin‐mediated proteasomal degradation presented by HSP70 under hyperglycemic conditions and further phosphorylates AKT. Moreover, the binding of FOXO3a with *Bim* was inhibited, resulting in apoptosis resistance (Figure 8(f)).

## 3 DISCUSSION

Accumulating evidence has highlighted the cytoprotective effects of umbilical cord stem cells against various diseases. Nevertheless, it has also been shown that under stressful conditions, stem cells display reduced potential. An increasing number of studies have shown the adverse effects of hyperglycemic conditions in different stem cells, including mesenchymal stem cells.^12^, ^13^, ^14^, ^38^ Taking these into consideration, we first evaluated the effect of HG on WJMSCs and the underlying mechanism involved in HG‐induced cellular injuries in WJMSCs. It was found that HG affects cell viability and affects the components of proteostasis machinery, such as HSP90, HSP70, and CHIP. Moreover, the effect of CHIP overexpression on WJMSCs under HG condition was elucidated further; interestingly, we found that HG activated PTEN and the downstream signaling cascade that ensued in the induction of apoptosis and oxidative stress via CHIP impairment in WJMSCs. Notably, it was found that CHIP targets and promotes proteasomal degradation of PTEN and in turn enhanced p‐AKT and p‐FOXO3a protein expression levels to inhibit HG‐induced apoptosis and oxidative stress. A previous study demonstrated that PTEN is activated during HG conditions and can induce severe cardiac complications, including oxidative stress and apoptosis, leading to diabetic cardiomyopathy.^39^ However, the underlying mechanism responsible for regulating PTEN is not fully understood in WJMSCs. It is well known that ubiquitin‐mediated proteasomal degradation plays an important role in protein quality control in order to maintain protein homeostasis.^40^ Ahmed et al. demonstrated that CHIP promotes the proteasomal degradation of PTEN.^41^ In addition, CHIP has the ability to target many proteins for proteasomal degradation.^42^, ^43^ Our results are consistent with previous studies, wherein it has been highlighted that CHIP is able to promote the ubiquitination and proteasomal degradation of PTEN, which may be supported by HSP70 and further promotes phosphorylation of AKT and FOXO3a to inhibit HG‐induced apoptosis and oxidative stress. Moreover, CHIP overexpression reduced the binding ability of FOXO3a with *Bim*. In addition, bioinformatics analysis confirmed the interaction of CHIP with PTEN. Docking results indicated that conserved amino‐acid residues play an important role in maintaining functional conformation and are directly involved in donor substrate binding. The interaction between CHIP and PTEN proposed in this study is useful for understanding the potential mechanism of domain and inhibitor binding. As is well known, hydrogen bonds play important roles in the structure and function of biological molecules, and we found that PRO102, GLN104, TRP105, and PHE106 in CHIP of *Homo sapiens* are important for strong hydrogen bonding interaction with THR147, GLY149, and ILE192 of PTEN. To the best of our knowledge, these are conserved in this domain and may be important for structural integrity or maintaining the hydrophobicity of the inhibitor‐binding pocket. Collectively, our study increased the understanding of the protective role of CHIP in regulating PTEN and the downstream signaling cascade triggered under HG conditions. As mentioned earlier, several studies demonstrated that FOXO3a, a vital transcription factor involved in many cellular processes, can bind to various promoter regions. Besides, it is well known that *bim*, a proapoptotic protein, can regulate apoptosis under several stress conditions.^44^, ^45^ Considering these, it was found that FOXO3a depletion downregulated *bim*, and similar results were obtained upon CHIP overexpression in WJMSCs, indicating that CHIP regulates binding of FOXO3a with the *bim* promoter region, which was further confirmed by docking studies. Cumulatively, the present study suggests that CHIP can modulate PTEN and the downstream signaling cascade and confers resistance to apoptosis by promoting PTEN proteasomal degradation.

Diabetes mellitus increases the risk of cardiovascular complications. Evidence has shown that hyperglycemia induces cardiomyopathies. Combining newer therapeutic strategies may provide hope for tackling the devastating complications associated with diabetes, both in the heart, as delineated here, and potentially in other organs as well. As a matter of fact, interest in stem cell‐based regenerative medicines has garnered great interest among the research fraternities. Interestingly, implementations of genetic engineering methodologies are capable of further enhancing the therapeutic potential of stem cells. Thus, our CHIP overexpressing WJMSCs may be an effective candidate in the frontiers of engineered stem cells with therapeutic potential against HG‐induced cardiac injury. Research has highlighted that genetically engineered stem cells have shown efficacy against various diseases.^46^, ^47^ Therefore, to ascertain their potential against HG‐induced cardiac complications, coculturing of CHIP overexpressing WJMSCs was performed. Interestingly, it was found that coculturing of WJMSCs with H9c2 rescued HG‐induced apoptosis and oxidative stress. Increasing our understanding may be instrumental in ameliorating stem cell effects and would certainly widen the horizon of stem cell therapeutics. This is indeed in agreement with other studies, wherein the authors have highlighted the potential of various engineered stem cells.^48^, ^49^, ^50^ Further, in vivo animal model experimentation was performed to explore the role of CHIP overexpressed WJMSCs against diabetes‐induced cardiac injury. Our in vitro findings are in line with the in vivo model, except that in the animal tissues WJMSCs alone exhibited some effect, which is inconsistent with the cell model findings. In conclusion, our research demonstrated the underlying intricacies regarding diabetes‐induced cellular injuries and provided evidence for the ameliorative effect of CHIP overexpressing WJMSCs against diabetes‐induced cardiac complications.

## 4 CONCLUSIONS

HG increased apoptosis and oxidative stress in cells with impaired proteostasis systems, which trigger the PTEN signaling cascade. CHIP, an E3 ligase, maintains PTEN under HG conditions via association with the chaperone system. Furthermore, CHIP knockdown stabilizes PTEN; while CHIP overexpression induces Akt and promotes the phosphorylation of FOXO3a, resulting in export from the nucleus to the cytoplasm, and inhibits the binding of FOXO3a with the *Bim* promoter. Moreover, the coculturing of CHIP overexpressing WJMSCs with H9c2 rescued HG‐induced apoptosis and oxidative stress, and its administration to STZ‐induced diabetic rats attenuated cardiac damage. Cumulatively, the present study reveals that CHIP overexpressing WJMSCs promote apoptosis resistance through alteration of PTEN and the downstream signaling cascade, and more specifically, by promoting PTEN proteasomal degradation. Further, CHIP overexpressing stem cells exert protective effects by inhibiting hyperglycemia‐induced cardiac damage in diabetic rats. Notably, the study highlights the therapeutic potential of CHIP overexpressing WJMSCs against diabetes‐induced cardiomyopathies.

## 5 MATERIALS AND METHODS

### 5.1 Animal model and experimental groups design

The experimental animal model performed was according to the NIH Guide for the Care and Use of Laboratory Animals. The protocols were approved by Institutional Animal Care and Use Committee of Hualien Tzu Chi hospital, Taiwan (IACUC approval no. 109‐02). Six‐week‐old SD rats of 230–255 g were acclimatized for 2 weeks in the core facility, and thereafter used for experiments. The rats used in this study were purchased from National Animal Breeding and Research Center (Taipei, Taiwan). All the rats were housed in individual cages at a constant temperature (22°C) on a 12‐h light–dark cycle with access to diet and water (Lab Diet 5001; PMI Nutrition International, Inc.). The experimental animals were administered with nicotinamide dissolved in 0.9% sodium chloride followed by STZ (55 mg/kg body weight after dissolved in citrate buffer with pH 4.5) via intraperitoneal cavity to induce diabetes. All the rats were arranged into six different groups: control SD rats (*n* = 9), STZ‐induced diabetes rats administered with streptozotocin injection (*n* = 9), STZ‐induced diabetes rats transplanted with WJMSCs (*n* = 9) (1 × 10^7^), STZ‐induced diabetic rats infused with GFP‐CHIP overexpressed WJMSCs (*n* = 9) (1 × 10^7^), STZ‐induced diabetes rats transplanted with WJMSCs containing shCHIP (*n* = 9) (1 × 10^7^), and STZ‐induced diabetic rats injected with shPTEN WJMSCs (*n* = 9) (1 × 10^7^). The WJMSCs alone, and the WJMSCs expressing lentiviral GFP‐CHIP, shCHIP, and shPTEN were injected twice via lateral tail vein.

### 5.2 Establishment of stable cell line

The lentiviral plasmids, including GFP‐CHIP, shCHIP, and shPTEN, were cotransfected with pMD.G and pCMVΔR8.91 plasmids in HEK 293T cell line. The medium was harvested from the 293T cells after 24 and 48 h post‐transfection. After the lentivirus packaging in the 293T, WJMSCs were infected using polybrene (10 μg/ml). After 48 h, the normal medium was replaced with the medium containing puromycin (5 μg/ml). Thereafter, the cells were harvested and used for experiments.

### 5.3 Oral glucose tolerance test

After 6 weeks treatment, OGTT was performed to assess insulin resistance. Briefly, rats were fasted for 14 h followed by glucose administration (2 g/kg body weight) using oral gavage method. Blood glucose was measured at the indicated time points (0, 30, 60, 90, 120) by tail vein pricking method using Accu‐Chek Guide blood glucose meter (Roche Diabetes Care).

### 5.4 Echocardiography

Echocardiography imaging was performed to evaluate the cardiac function following the instructions issued by American Society of Echocardiography using a 5–8 MHz sector and 12 MHz linear transducer (Vivid 3, General Electric Medical Systems Ultrasound). Briefly, rats were anesthetized, and M‐mode as well as two dimensional images were obtained in the parasternal long and short axes. The cardiac parameters, including LVD, interventricular septal thickness, and left ventricular posterior wall thickness, were obtained during systole (s) and diastole (d). EF and FS were based on the values obtained from echocardiography images.

### 5.5 Immunohistochemical staining

As mentioned above, the cardiac tissue sections were deparaffinized with xylene and rehydrated using graded series of alcohol followed by permeabilization, blocking, and washed with phosphate‐buffered saline (PBS). Then, the tissue slides were probed with the respective primary antibody for 1 h, washed with PBS, and incubated with the horseradish peroxidase (HRP)‐conjugated avidin biotin complex using Vectastain Elite ABC Kit and NovaRED chromogen (Vector Laboratories) followed by hematoxylin stain. Expression of cardiac PTEN and FOXO3a was measured using microscopy (OLYMPUS® BX53).

### 5.6 Reagents and antibodies

All chemicals and reagents were procured from Sigma Aldrich (Sigma Aldrich) unless and otherwise mentioned. Plasmid with the backbone of pRK5 expressing HA tag encoding CHIP was gifted from Dr. Jeng‐Fan Lo (National Yang‐Ming Medical University, Taipei, Taiwan). Lentiviral GFP‐CHIP was purchased from the Sino biological (RG83573‐ACGLN), while the lentiviral expressing small hairpin RNAs (shRNAs), including shcontrol (pLAS.Void), shCHIP (TRCN0000007528 NM_005861), shPTEN (TRCN0000002746 NM_000314), and lentiviral packaging plasmids (pCMVΔR8.91 and pMD.G), were obtained from the national RNAi core facility (Academia Sinica). PTEN inhibitor (VO‐OHpic trihydrate, sc‐216,061) was purchased from Santa Cruz Biotechnology.

The primary antibodies used in this study are: anti‐CHIP (sc‐66,830), anti‐Bcl‐2 (C‐2) (sc‐7382), anti‐Bax (P‐19) (sc‐526), anti‐Bad (C‐7) (sc‐8044), anti‐cytochrome C (7H8) (sc‐13,560), anti‐Bcl‐xL (H‐5) (sc‐8392), anti‐NOX‐2/gp91 phox (sc‐5827), anti‐p47^phox^ (sc‐14,015), anti‐p22^phox^ (FL‐195) (sc‐20,781), anti‐SOD‐2 (MnS‐1) (sc‐65,437), anti‐catalase (H‐9) (sc‐271,803) anti‐Akt1 (B‐1) (sc‐5298), anti‐p‐Akt1/2/3 (Ser473) (sc‐7985), anti‐Bim (H‐5) (sc‐374,358), anti‐HA (sc‐7392), anti‐GFP (FL) (sc‐8334), anti‐ubiquitin (sc‐8017), anti‐HDAC1 (sc‐81,598), anti‐β‐actin (sc‐47,778), anti‐GAPDH (6C5) (sc‐32,233) (Santa Cruz Biotechnology), anti‐PTEN (#9559s), anti‐FOXO3a (75D8) (#2497), anti‐p‐FOXO3a (Ser253) (#9466), anti‐HSP70 (#4872), anti‐HSP90 (#4877) (Cell Signaling Technology), anti‐Rac1 (ab33186, Abcam), anti‐NEDD4 (A0552), anti‐XIAP (A6869), and anti‐WWP2 (A2425, ABclonal). The secondary antibodies against goat, mouse, and rabbit conjugated with HRP were purchased from Santa Cruz Biotechnology.

### 5.7 Cell culture, transient transfection, and gene silencing

WJMSCs purchased from Bioresource Collection and Research Center (BCRC) were grown and maintained in 5% CO_2_ humidified incubator (Thermo Fisher Scientific) at 37°C in Dulbecco's modified Eagle's medium (DMEM) supplemented with 10% fetal bovine serum (FBS) (HyClone), 2 mM glutamine, 1.5 g/L sodium bicarbonate, 100 U/ml penicillin, and 100 mg/ml streptomycin. Briefly, WJMSCs with 50%–70% confluency were challenged with HG (40 mM) for 24 h, followed by plasmids and/or siRNA transfection for 24 h using JetPrime transfection reagent (Polyplus‐transfection) according to the manufacturer instructions. In this study, MG‐132 (proteasome inhibitor), cycloheximide (CHX) (protein synthesis inhibitor), VO‐OHpic trihydrate (PTEN inhibitor), and LY294002 (PI3K inhibitor) were treated in the presence of HG.

### 5.8 Western blotting and immunoprecipitation

Western blot analysis was performed as described in our recent studies.^51^, ^52^ In brief, WJMSCs were centrifuged at 13,000*g* for 20 min after lysed with lysis buffer (50 mM Tris‐base, 1 M EDTA, 0.5 M NaCl, 1 mM beta‐mercaptoethanol, 1% NP‐40, protease inhibitor tablet [Roche] and 10% glycerol). Thereafter, total cell extract was quantified using Bradford assay (Bio‐Rad), separated by 10%–12% sodium dodecyl sulfate polyacrylamide gel electrophoresis (SDS‐PAGE), and then transferred to a Polyvinylidene fluoride (PVDF) membrane (Millipore). Then, membrane was blocked for 1 h in 5% blocking buffer (skim‐milk) followed by overnight incubation in primary antibodies at 4°C. In the next step, membrane was incubated with secondary antibodies (1:3000 dilution) conjugated with HRP for 1 h at room temperature (RT). Finally, the analysis was obtained using enhanced chemiluminescence (ECL) kit (Millipore), and visualized with LAS 3000 imaging system (Fujifilm). All the images were quantified and analyzed using ImageJ (NIH) and GraphPad prism5 software, respectively.

Whole cell lysates from the WJMSCs were immunoprecipitated using the Protein G magnetic beads (Millipore) following the manufacturer's guidelines. A total of 500 μg protein lysates were incubated with the 2 μg of respective primary antibody overnight on a rotator at 4°C. Immunoprecipitated proteins were eluted at 95°C and thereafter separated using SDS‐PAGE followed by transfer to a PVDF membrane, and probed with specific primary antibody.

### 5.9 Cell viability assay

A colorimetric assay was performed to estimate the cell viability on the principle of conversion tetrazolium (MTT) dye (3‐4,5‐dimethylthiazol‐2‐yl)‐2,5‐diphenyltetrazolium‐bromide) into a formazan product with blue color formation. After harvest, cells were washed twice with PBS and cultured in DMEM (1 ml) with 10% FBS. MTT (0.5 mg/ml) was added to cells for 4 h and kept at 37°C. Cell viability was measured at optical density (OD) 570 nm spectrophotometrically after incubation and shaking for 10 min in dimethyl sulfoxide (DMSO).

### 5.10 Subcellular fractionation

The cytoplasmic and nuclear extracts were obtained after transfection with siCHIP in the presence of HG stress using Nuclear and Cytosol fractionation kit (BioVision) following the manufacturer's instructions. Briefly, 30–40 μg of separated proteins were analyzed via immunoblotting according to the standard described method.

### 5.11 Detection of mitochondrial ROS

Mitochondrial superoxide generation was measured in WJMSCs and H9c2 cells using MitoSOX (Invitrogen Molecular Probes). After WJMSCs were transfected and challenged with HG for 24 h, cells were incubated with MitoSOX for 30 min at 37°C, followed by 4′,6‐diamidino‐2‐phenylindole (DAPI) for 5 min to examine the cell nuclei. Mitochondrial ROS generation was measured using fluorescence microscopy (Olympus), with the excitation and emission wavelength in the range of 510/580 nm.

### 5.12 TUNEL assay

After CHIP plasmid transfection and challenged with HG, cells were fixed with 4% Paraformaldehyde for 1 h at RT. After washing with PBS, cells were permeabilized with Triton X‐100 (0.1%) in sodium citrate (0.1%), and incubated with TUNEL reagent to measure apoptosis using apoptotic detection kit (Roche Diagnostic). In cardiac tissue, slides were deparaffinized, rehydrated followed by incubation with 3% H_2_O_2_. Thereafter, sections were washed, and incubated with TUNEL reagent for 1 h at 37°C. Next, cells were incubated with DAPI for 5 min, followed by washing with PBS. Finally, apoptosis was examined by detecting the TUNEL‐positive cells using fluorescence microscopy (Olympus) having excitation and emission wavelength of 450–500 nm and 515–565 nm, respectively. The number of TUNEL‐positive cells counted manually and statistically analyzed using GraphPad Prism5 software.

### 5.13 Flow cytometry for apoptosis detection

Cells transfected with HA‐vector, HA‐CHIP, shcontrol, and/or shCHIP plasmid in the presence of HG were harvested, and washed twice with PBS. Then, cells were resuspended in 1x binding buffer, and incubated for 15–30 min with fluorescein isothiocyanate (FITC) annexin V fluorescein and propidium iodide dye using FITC Annexin V detection kit (BD Biosciences) following the manufacturer instructions and analyzed by fluorescence activated cell sorting (BD Biosciences). The statistical analyses were based on the 10,000 cells per event.

### 5.14 Chromatin immunoprecipitation

ChIP assay was performed to assess the probing of protein‐DNA complex. SimpleChIP Enzymatic Chromatin IP kit (Magnetic beads, #9003) was procured from Cell Signaling Technology. ChIP assay was performed following the guidelines provided by manufacturer. Briefly, WJMSCs transfected with HA‐CHIP or shCHIP under HG conditions were subjected to chromatin digestion and immunoprecipitated with anti‐FOXO3a and Normal Rabbit IgG antibody. Further, the precipitated DNA was amplified by quantitative real‐time polymerase chain reaction (QuantStudio 5 Applied Biosystems) using the power SYBR™ Green PCR Master Mix (Applied Biosystems). Primers used in the study to amplify the *bim* promoter region containing the FOXO3a binding sites are following. *Bim* forward: 5′‐AGGCAGAACAGGAGGAGA‐3′; Bim reverse: 5′‐AACCCGTTTGTAAGAGGC‐3′.

### 5.15 Coculturing

WJMSCs and H9c2 cardiomyocytes were cocultured in six‐well Transwell inserts (Corning) with 0.4 μm pore size, and maintained in a 5% CO_2_ humidified incubator. WJMSCs alone, WJMSCs expressing HA‐CHIP, and HA‐vector were seeded in the inner transwell chamber, while cardiac cells in the lower chamber were challenged with HG.

### 5.16 Statistical analysis

Results are shown as mean ± SD. Statistical analysis was performed using GraphPad Prism5 statistical software. Multiple comparisons were accessed through one‐way analysis of variance (ANOVA) and *p* value of <0.05 was considered statistically significant.

## CONFLICT OF INTEREST

The authors have no conflicts of interest to declare.

## AUTHOR CONTRIBUTIONS

Ayaz Ali: conceptualization, data curation, formal analysis, investigation, writing—original draft, writing—review and editing. Wei‐Wen Kuo: data curation, formal analysis, investigation, methodology, supervision, review and editing. Chia‐Hua Kuo: conceptualization, data curation, formal analysis, visualization, software. Jeng‐Fan Lo: project administration, resources, software. Michael Y. C. Chen: supervision, validation, visualization. Jayasimha R. Daddam: supervision, validation, visualization. Tsung‐Jung Ho: project administration, resources, software. Viswanadha Vijaya Padma: supervision, administration, resources, software. Marthandam Asokan Shibu: methodology, project administration, resources, supervision, funding acquisition. Chih‐Yang Huang: conceptualization, data curation, funding acquisition, investigation, administration, resources, supervision, validation. All authors revised the manuscript critically for important intellectual content and approved the final version of the manuscript.

### PEER REVIEW

The peer review history for this article is available at https://publons.com/publon/10.1002/btm2.10234.

## Supporting information

**Appendix S1**: Supporting Information

## Data Availability

All data generated or analyzed during this study are included in this article.

## References

[1] Einarson TR, Acs A, Ludwig C. Prevalence of cardiovascular disease in type 2 diabetes: a systematic literature review of scientific evidence from across the world in 2007–2017. Cardiovasc Diabetol. 2018;17(1):83. 29884191 10.1186/s12933-018-0728-6 PMC5994068

[2] Abdul‐Ghani M, DeFronzo RA, Del Prato S, et al. Cardiovascular disease and type 2 diabetes: has the dawn of a new era arrived? Diabetes Care. 2017;40:813‐820. 28637886 10.2337/dc16-2736 PMC5481984

[3] Filardi T, Ghinassi B. Cardiomyopathy associated with diabetes: the central role of the cardiomyocyte. Int J Mol Sci. 2019;20(13):3299. 10.3390/ijms20133299 PMC6651183 31284374

[4] Lyons TJ, Basu A. Biomarkers in diabetes: hemoglobin A1c, vascular and tissue markers. Transl Res. 2012;159(4):303‐312. 22424433 10.1016/j.trsl.2012.01.009 PMC3339236

[5] Yang YC, Tsai CY, Chen CL, et al. Pkcδ activation is involved in ROS‐mediated mitochondrial dysfunction and apoptosis in cardiomyocytes exposed to advanced glycation end products (ages). Aging Dis. 2018;9(4):647‐663. 30090653 10.14336/AD.2017.0924 PMC6065295

[6] Nagy T, Fisi V, Frank D, et al. Hyperglycemia‐induced aberrant cell proliferation; a metabolic challenge mediated by protein O‐GlcNAc modification. Cells. 2019;8(9):999. 10.3390/cells8090999 PMC6769692 31466420

[7] Huang YT, Yao CH, Way CL, et al. Diallyl trisulfide and diallyl disulfide ameliorate cardiac dysfunction by suppressing apoptotic and enhancing survival pathways in experimental diabetic rats. J Appl Physiol. 2013;114(3):402‐410. 23139364 10.1152/japplphysiol.00672.2012

[8] Morresi C, Cianfruglia L, Sartini D, et al. Effect of high glucose‐induced oxidative stress on paraoxonase 2 expression and activity in Caco‐2 cells. Cells. 2019;8(12):1616. 10.3390/cells8121616 PMC6953021 31835890

[9] Jia G, Hill MA, Sowers JR. Diabetic cardiomyopathy: an update of mechanisms contributing to this clinical entity. Circ Res. 2018;122(4):624‐638. 29449364 10.1161/CIRCRESAHA.117.311586 PMC5819359

[10] Borghetti G, von Lewinski D, Eaton DM, et al. Diabetic cardiomyopathy: current and future therapies. Beyond glycemic control. Front Physiol. 2018;9:1514. 30425649 10.3389/fphys.2018.01514 PMC6218509

[11] Tan Y, Zhang Z. Mechanisms of diabetic cardiomyopathy and potential therapeutic strategies: preclinical and clinical evidence. Nat Rev Cardiol. 2020;17(9):585‐607. 32080423 10.1038/s41569-020-0339-2 PMC7849055

[12] Cheng NC, Hsieh TY, Lai HS, Young TH. High glucose‐induced reactive oxygen species generation promotes stemness in human adipose‐derived stem cells. Cytotherapy. 2016;18(3):371‐383. 26780864 10.1016/j.jcyt.2015.11.012

[13] Hankamolsiri W, Manochantr S, Tantrawatpan C, et al. The effects of high glucose on adipogenic and osteogenic differentiation of gestational tissue‐derived MSCs. Stem Cells Int. 2016;2016:9674614. 27057179 10.1155/2016/9674614 PMC4707328

[14] Wajid N, Naseem R, Anwar SS, et al. The effect of gestational diabetes on proliferation capacity and viability of human umbilical cord‐derived stromal cells. Cell Tissue Bank. 2015;16(3):389‐397. 25407535 10.1007/s10561-014-9483-4

[15] Khan M, Ali F, Mohsin S, et al. Preconditioning diabetic mesenchymal stem cells with myogenic medium increases their ability to repair diabetic heart. Stem Cell Res Ther. 2013;4(3):58. 23706645 10.1186/scrt207 PMC3707006

[16] Bernardi S, Severini GM, Zauli G, Secchiero P. Cell‐based therapies for diabetic complications. Exp Diabetes Res. 2012;2012:872504. 21822425 10.1155/2012/872504 PMC3123995

[17] Musial‐Wysocka A, Kot M. Molecular and functional verification of Wharton's jelly mesenchymal stem cells (WJ‐MSCs) pluripotency. Int J Mol Sci. 2019;20(8):1807. 10.3390/ijms20081807 PMC6515095 31013696

[18] Witkowska‐Zimny M, Wrobel E. Perinatal sources of mesenchymal stem cells: Wharton's jelly, amnion and chorion. Cell Mol Biol Lett. 2011;16(3):493‐514. 21786036 10.2478/s11658-011-0019-7 PMC6275796

[19] Kim DW, Staples M, Shinozuka K, Pantcheva P, Kang SD, Borlongan C. Wharton's jelly‐derived mesenchymal stem cells: phenotypic characterization and optimizing their therapeutic potential for clinical applications. Int J Mol Sci. 2013;14(6):11692‐11712. 23727936 10.3390/ijms140611692 PMC3709752

[20] Cutler AJ, Limbani V, Girdlestone J, Navarrete CV. Umbilical cord‐derived mesenchymal stromal cells modulate monocyte function to suppress T cell proliferation. J Immunol. 2010;185(11):6617‐6623. 20980628 10.4049/jimmunol.1002239

[21] Wang Y, Chen X, Cao W, Shi Y. Plasticity of mesenchymal stem cells in immunomodulation: pathological and therapeutic implications. Nat Immunol. 2014;15(11):1009‐1016. 25329189 10.1038/ni.3002

[22] Birnbaum Y, Nanhwan MK, Ling S, Perez‐Polo JR, Ye Y, Bajaj M. PTEN upregulation may explain the development of insulin resistance and type 2 diabetes with high dose statins. Cardiovasc Drugs Ther. 2014;28(5):447‐457. 25106875 10.1007/s10557-014-6546-5

[23] Shen Y, Zhang J, Yu T, Qi C. Generation of PTEN knockout bone marrow mesenchymal stem cell lines by CRISPR/Cas9‐mediated genome editing. Cytotechnology. 2018;70(2):783‐791. 29387984 10.1007/s10616-017-0183-3 PMC5851970

[24] Ye X, Wang L, Shang B, Wang Z, Wei W. NEDD4: a promising target for cancer therapy. Curr Cancer Drug Targets. 2014;14(6):549‐556. 25088038 10.2174/1568009614666140725092430 PMC4302323

[25] Wang X, Trotman LC, Koppie T, et al. NEDD4‐1 is a proto‐oncogenic ubiquitin ligase for PTEN. Cell. 2007;128(1):129‐139. 17218260 10.1016/j.cell.2006.11.039 PMC1828909

[26] Tokuhira N, Kitagishi Y, Suzuki M, et al. PI3K/AKT/PTEN pathway as a target for Crohn's disease therapy (review). Int J Mol Med. 2015;35(1):10‐16. 25352295 10.3892/ijmm.2014.1981

[27] Vallee A, Lecarpentier Y, Guillevin R, Vallee JN. Interactions between TGF‐beta1, canonical WNT/beta‐catenin pathway and PPAR gamma in radiation‐induced fibrosis. Oncotarget. 2017;8(52):90579‐90604. 29163854 10.18632/oncotarget.21234 PMC5685775

[28] Butler M, McKay RA, Popoff IJ, et al. Specific inhibition of PTEN expression reverses hyperglycemia in diabetic mice. Diabetes. 2002;51(4):1028‐1034. 11916922 10.2337/diabetes.51.4.1028

[29] Hu Z, Lee IH, Wang X, et al. PTEN expression contributes to the regulation of muscle protein degradation in diabetes. Diabetes. 2007;56(10):2449‐2456. 17623817 10.2337/db06-1731

[30] Drinjakovic J, Jung H, Campbell DS, Strochlic L, Dwivedy A, Holt CE. E3 ligase Nedd4 promotes axon branching by downregulating PTEN. Neuron. 2010;65(3):341‐357. 20159448 10.1016/j.neuron.2010.01.017 PMC2862300

[31] Lamb JR, Tugendreich S, Hieter P. Tetratrico peptide repeat interactions: to TPR or not to TPR? Trends Biochem Sci. 1995;20(7):257‐259. 7667876 10.1016/s0968-0004(00)89037-4

[32] Ye Z, Needham PG, Estabrooks SK, et al. Symmetry breaking during homodimeric assembly activates an E3 ubiquitin ligase. Sci Rep. 2017;7(1):1789. 28496195 10.1038/s41598-017-01880-4 PMC5431976

[33] VanPelt J, Page RC. Unraveling the CHIP:Hsp70 complex as an information processor for protein quality control. Biochim Biophys Acta Proteins Proteomics. 2017;1865(2):133‐141. 27863257 10.1016/j.bbapap.2016.11.005

[34] Wang L, Zhang TP, Zhang Y, et al. Protection against doxorubicin‐induced myocardial dysfunction in mice by cardiac‐specific expression of carboxyl terminus of hsp70‐interacting protein. Sci Rep. 2016;6:28399. 27323684 10.1038/srep28399 PMC4914971

[35] Huang CY, Kuo WW, Lo JF, et al. Doxorubicin attenuates CHIP‐guarded HSF1 nuclear translocation and protein stability to trigger IGF‐IIR‐dependent cardiomyocyte death. Cell Death Dis. 2016;7(11):e2455. 27809308 10.1038/cddis.2016.356 PMC5260882

[36] Feng CC, Liao PH, Tsai HI, et al. Tumorous imaginal disc 1 (TID1) inhibits isoproterenol‐induced cardiac hypertrophy and apoptosis by regulating c‐terminus of hsc70‐interacting protein (CHIP) mediated degradation of Gαs. Int J Med Sci. 2018;15(13):1537‐1546. 30443176 10.7150/ijms.24296 PMC6216068

[37] Wang Z, Li Y, Wang Y, Zhao K, Chi Y, Wang B. Pyrroloquinoline quinine protects HK‐2 cells against high glucose‐induced oxidative stress and apoptosis through Sirt3 and PI3K/Akt/FoxO3a signaling pathway. Biochem Biophys Res Commun. 2019;508(2):398‐404. 30502093 10.1016/j.bbrc.2018.11.140

[38] Cianfarani F, Toietta G, Di Rocco G, et al. Diabetes impairs adipose tissue‐derived stem cell function and efficiency in promoting wound healing. Wound Repair Regen. 2013;21(4):545‐553. 23627689 10.1111/wrr.12051

[39] Song P, Wu Y, Xu J, et al. Reactive nitrogen species induced by hyperglycemia suppresses Akt signaling and triggers apoptosis by upregulating phosphatase PTEN (phosphatase and tensin homologue deleted on chromosome 10) in an LKB1‐dependent manner. Circulation. 2007;116(14):1585‐1595. 17875968 10.1161/CIRCULATIONAHA.107.716498

[40] Ciechanover A, Kwon YT. Protein quality control by molecular chaperones in neurodegeneration. Front Neurosci. 2017;11:185. 28428740 10.3389/fnins.2017.00185 PMC5382173

[41] Ahmed SF, Deb S, Paul I, et al. The chaperone‐assisted E3 ligase C terminus of Hsc70‐interacting protein (CHIP) targets PTEN for proteasomal degradation. J Biol Chem. 2012;287(19):15996‐16006. 22427670 10.1074/jbc.M111.321083 PMC3346122

[42] Xie P, Fan Y, Zhang H, et al. CHIP represses myocardin‐induced smooth muscle cell differentiation via ubiquitin‐mediated proteasomal degradation. Mol Cell Biol. 2009;29(9):2398‐2408. 19237536 10.1128/MCB.01737-08 PMC2668377

[43] Xu CW, Zhang TP, Wang HX, Yang H, Li HH. CHIP enhances angiogenesis and restores cardiac function after infarction in transgenic mice. Cell Physiol Biochem. 2013;31(2‐3):199‐208. 23485987 10.1159/000343361

[44] Dong L, Vaux DL. Glucocorticoids can induce BIM to trigger apoptosis in the absence of BAX and BAK1. Cell Death Dis. 2020;11(6):442. 32513923 10.1038/s41419-020-2599-5 PMC7280233

[45] Mailleux AA, Overholtzer M, Schmelzle T, Bouillet P, Strasser A, Brugge JS. BIM regulates apoptosis during mammary ductal morphogenesis, and its absence reveals alternative cell death mechanisms. Dev Cell. 2007;12(2):221‐234. 17276340 10.1016/j.devcel.2006.12.003 PMC2698712

[46] Jeon SH, Zhu GQ. Engineered mesenchymal stem cells expressing stromal cell‐derived factor‐1 improve erectile dysfunction in streptozotocin‐induced diabetic rats. Int J Mol Sci. 2018;19(12):3730. 10.3390/ijms19123730 PMC6321323 30477146

[47] Ben Nasr M, Tezza S. PD‐L1 genetic overexpression or pharmacological restoration in hematopoietic stem and progenitor cells reverses autoimmune diabetes. Sci Transl Med. 2017;9(416):eaam7543. 29141886 10.1126/scitranslmed.aam7543 PMC6171337

[48] Ryu S, Lee JM, Bae CA, Moon CE, Cho KO. Therapeutic efficacy of neuregulin 1‐expressing human adipose‐derived mesenchymal stem cells for ischemic stroke. PLoS One. 2019;14(9):e0222587. 31560696 10.1371/journal.pone.0222587 PMC6764745

[49] Shen H, Cui G, Li Y, et al. Follistatin‐like 1 protects mesenchymal stem cells from hypoxic damage and enhances their therapeutic efficacy in a mouse myocardial infarction model. Stem Cell Res Ther. 2018;10(1):17. 10.1186/s13287-018-1111-y PMC6330478 30635025

[50] Kim SW, Lee DW, Yu LH, et al. Mesenchymal stem cells overexpressing GCP‐2 improve heart function through enhanced angiogenic properties in a myocardial infarction model. Cardiovasc Res. 2012;95(4):495‐506. 22886775 10.1093/cvr/cvs224

[51] Wu KM, Hsu YM, Ying MC, et al. High‐density lipoprotein ameliorates palmitic acid‐induced lipotoxicity and oxidative dysfunction in H9c2 cardiomyoblast cells via ROS suppression. Nutr Metab. 2019;16:36. 10.1186/s12986-019-0356-5 PMC6537189 31149020

[52] Liu SC, Tsai CH, Wu TY, et al. Soya‐cerebroside reduces IL‐1β‐induced MMP‐1 production in chondrocytes and inhibits cartilage degradation: implications for the treatment of osteoarthritis. Food Agric Immunol. 2019;30(1):620‐632.

