# Supplementary material for: E3 ligase activity of Carboxyl terminus of Hsc70 interacting protein (CHIP) in Wharton's jelly derived mesenchymal stem cells improves their persistence under hyperglycemic stress and promotes the prophylactic effects against diabetic cardiac damages
Source: Bioeng Transl Med. 2021 Jun 11;6(3):e10234. doi: 10.1002/btm2.10234 (PMC8459600; doi:10.1002/btm2.10234)
Supplement: Supplementary file 1 — Appendix S1: Supporting Information [file BTM2-6-e10234-s001.docx]

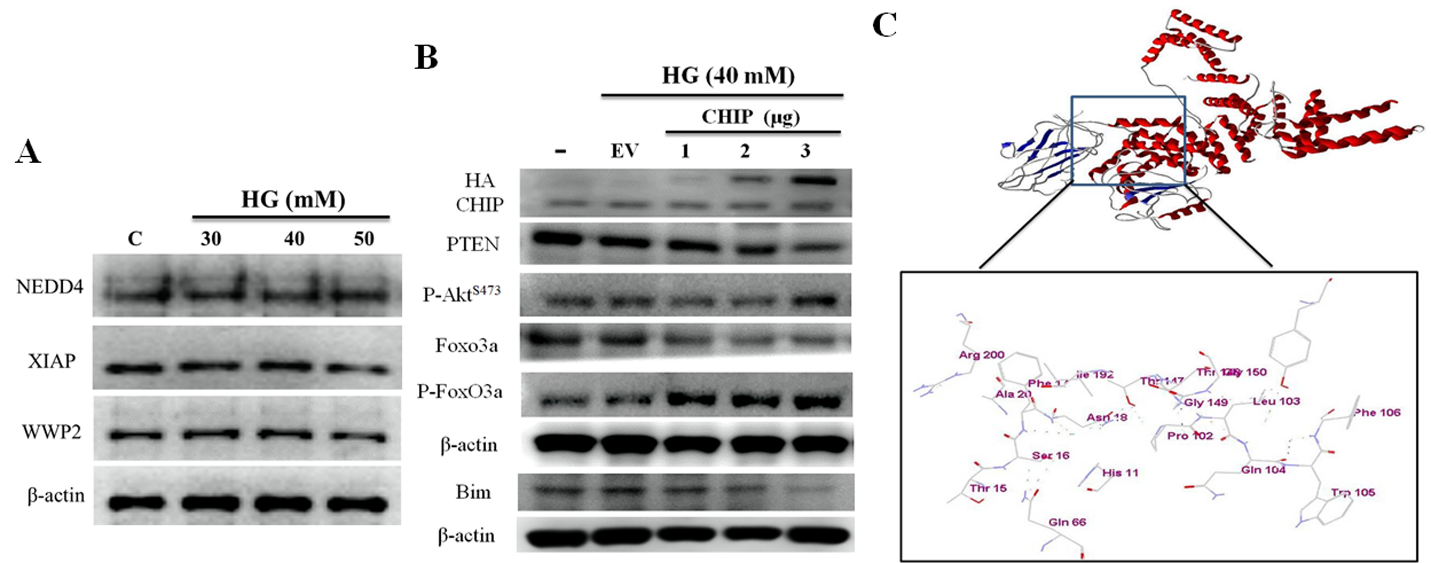


**Fig S2. Molecular docking studies of CHIP with PTEN (A)** WJMSCs challenged with increasing concentrations of HG (30, 40, and 50 mM) for 24 h were subjected to western blot analysis to evaluate the protein expression of NEDD4, XIAP, and WWP2. **(B)** WJMSCs were transfected with increasing concentrations of pRK5-HA-CHIP plasmid (1, 2, and 3 µg) in the presence of HG (40 mM) for 24 h, and the protein expression levels were analyzed via immunoblotting. (**C**) Molecular docking of CHIP with PTEN; the hydrogen bonding interactions and amino acids involved in docking were predicted.


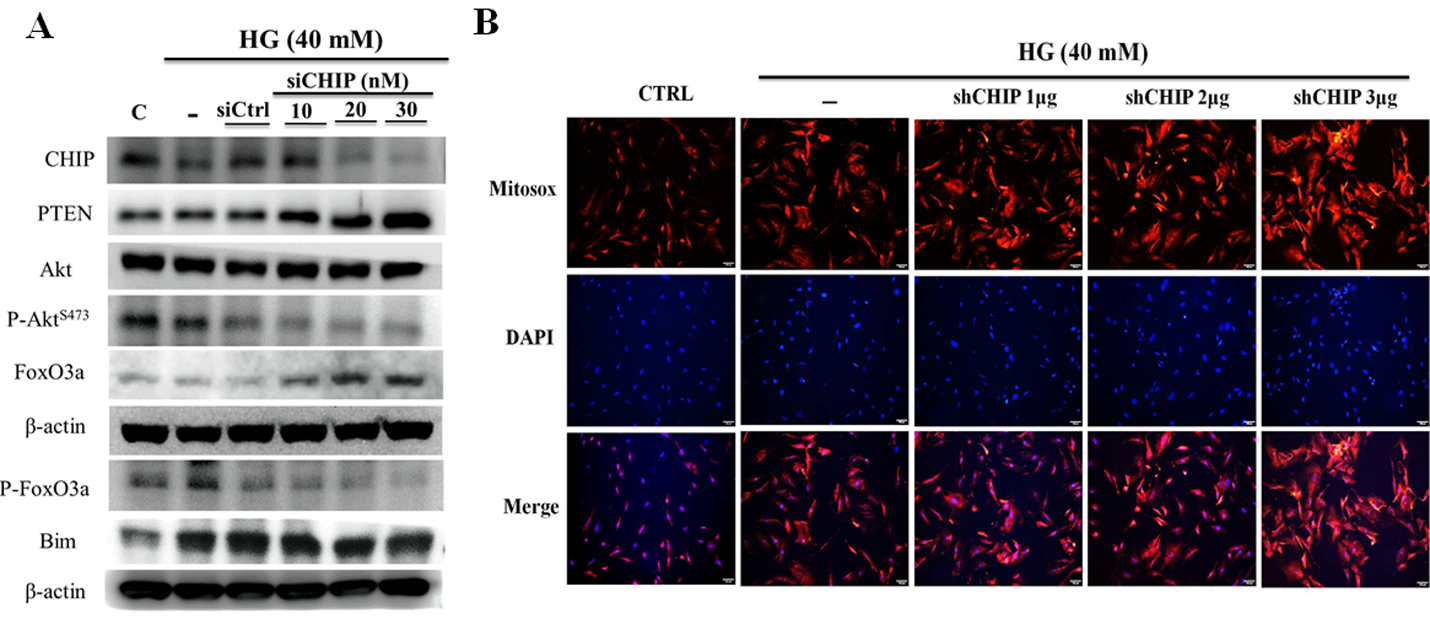


**Fig S3. CHIP regulates PTEN and its downstream signaling mediators under HG conditions. (A)** WJMSCs transfected with either sicontrol or increasing concentration of siCHIP followed by HG incubation for 24 h were immunoblotted, and the protein expression of PTEN and the downstream signaling mediators were assessed. **(B)** WJMSCs transfected with shcontrol or increasing amount of shCHIP plasmid (1, 2, and 3 µg) in the presence of HG for 24 h were analyzed for mitochondrial ROS generation.

**
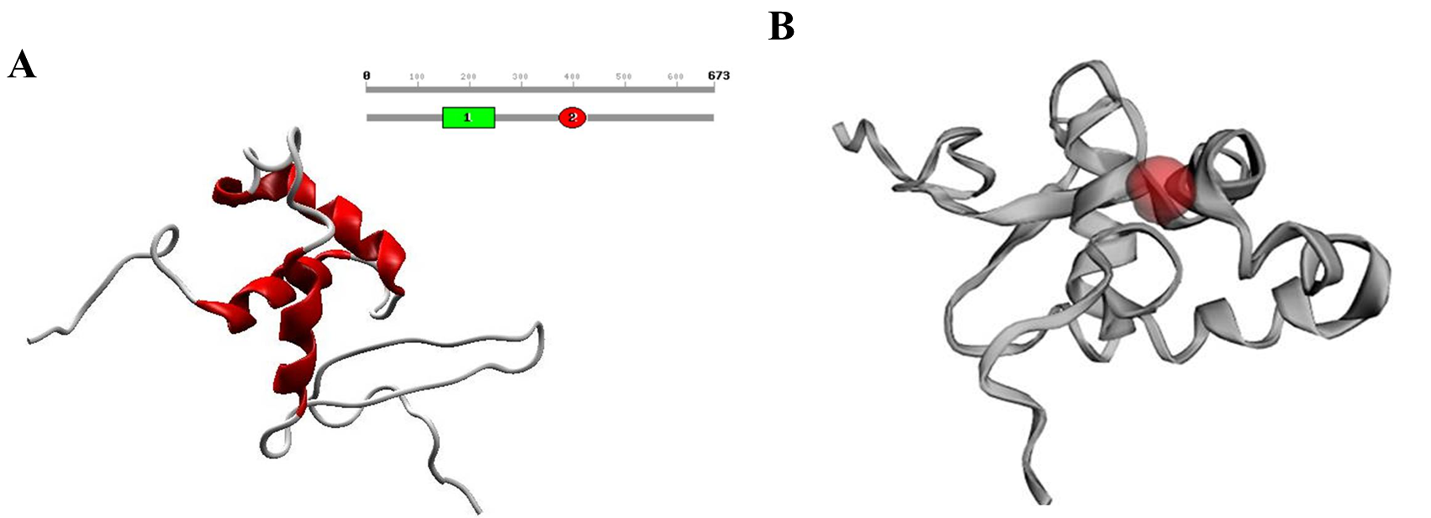
**

**Fig S5. Insilico analysis of binding FOXO3a with the *bim* promoter region** **(A)** The three dimensional structure of FoxO3a with three helices; two different domains fork head transcription factor (1); and unknown function (2). **(B)** Prediction of binding sites for FOXO3a using Castp server (red color).

**
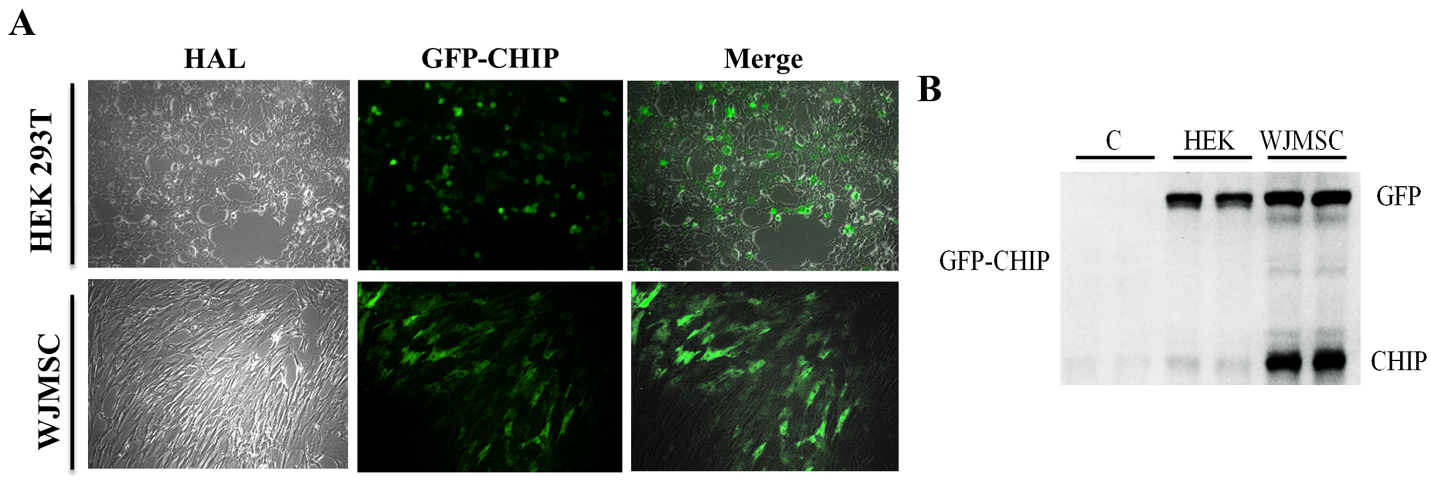
**

**Fig S7. Establishment of stable cell line** **(A)** Lentiviral particles from HEK293T cells were transduced into WJMSCs for 24 and 48 h, and examined under fluorescence microscope. **(B)** Total cell lysate from HEK293T and WJMSCs was harvested and immunoblotted to analyze the expression of GFP-CHIP.

**Materials and Methods**

**Hematoxylin and eosin (HE), Masson’s trichrome, (MT) and Periodic acid–Schiff staining (PAS)**

The tissues slides were deparaffinized using xylene followed by rehydration via gradient alcohol series. All the tissue sections were incubated with HE, MT, and PAS staining dye and subsequently washed with the water. Then, the animal tissues were dehydrated using gradient alcohol series, soaked in xylene, and mounted. Finally, images were obtained using microscopy (OLYMPUS® BX53, Tokyo, Japan).

**Insilico analysis**

The CHIP and PTEN sequences from *Homo sapiens* were submitted to SBASE server (<http://pongor.itk.ppke.hu/protein/sbase.html#/sbase>) for domain prediction and structures were collected from PDB database (<https://www.rcsb.org/>). Active site of PTEN and CHIP were identified using CASTp server (<http://sts.bioe.uic.edu/castp>). CHIP is docked into the active site of PTEN, and the interaction of CHIP with the active site residues are thoroughly studied using calculations of molecular mechanics using GOLD 3.0.1 software. The default algorithm speed was selected, and the inhibitor binding site in PTEN was defined within a 10Å radius with the centroid as HH atom of SER94 in *Homo sapiens* respectively. After docking, the individual binding poses of CHIP was observed and interaction with the PTEN were studied. The best and most energetically favorable conformation of CHIP was selected ^1^.

*Bim* promoter region was collected from NCBI database, (<https://www.ncbi.nlm.nih.gov/>) and drawn using Avogadro software which was a molecule generator algorithm. Later the FOXO3a structure collected from the PDB database was docked with *Bim* promoter sequence using GOLD 3.0.1. The binding studies of *Bim* promoter with FOXO3a protein predicted to find the interaction sites of FOXO3a with the *Bim* promoter region ^2^.

1. Daddam JR, Dowlathabad MR, Panthangi S, Jasti P. Molecular docking and P-glycoprotein inhibitory activity of flavonoids. *Interdiscip Sci.* 2014;6(3):167-175.

2. Kurjogi M, Satapute P, Jogaiah S, et al. Computational Modeling of the Staphylococcal Enterotoxins and Their Interaction with Natural Antitoxin Compounds. *Int j mol sci.* 2018;19(1).
